# Poly(lactic acid) (PLA) and polyhydroxyalkanoates (PHAs), green alternatives to petroleum-based plastics: a review

**DOI:** 10.1039/d1ra02390j

**Published:** 2021-05-10

**Authors:** Ahmed Z. Naser, I. Deiab, Basil M. Darras

**Affiliations:** Advanced Manufacturing Laboratory, University of Guelph Guelph ON Canada anaser@uoguelph.ca ideiab@uoguelph.ca; Department of Mechanical Engineering, American University of Sharjah Sharjah UAE bdarras@aus.edu

## Abstract

In spite of the fact that petroleum-based plastics are convenient in terms of fulfilling the performance requirements of many applications, they contribute significantly to a number of ecological and environmental problems. Recently, the public awareness of the negative effects of petroleum-based plastics on the environment has increased. The present utilization of natural resources cannot be sustained forever. Furthermore, oil is often subjected to price fluctuations and will eventually be depleted. The increase in the level of carbon dioxide due to the combustion of fossil fuel is causing global warming. Concerns about preservation of natural resources and climate change are considered worldwide motivations for academic and industrial researchers to reduce the consumption and dependence on fossil fuel. Therefore, bio-based polymers are moving towards becoming the favorable option to be utilized in polymer manufacturing, food packaging, and medical applications. This paper represents an overview of the feasibility of both Poly Lactic Acid (PLA) and polyhydroxyalkanoates (PHAs) as alternative materials that can replace petroleum-based polymers in a wide range of industrial applications. Physical, thermal, rheological, and mechanical properties of both polymers as well as their permeability and migration properties have been reviewed. Moreover, PLA's recyclability, sustainability, and environmental assessment have been also discussed. Finally, applications in which both polymers can replace petroleum-based plastics have been explored and provided.

## Abstract 2

The dwindling nature, high price of petroleum, concerns about climate change, as well as the ever-growing population are all urging the plastics industries to adapt sustainable natural biopolymers solutions such as PLA and PHAs.

## Graphical abstract

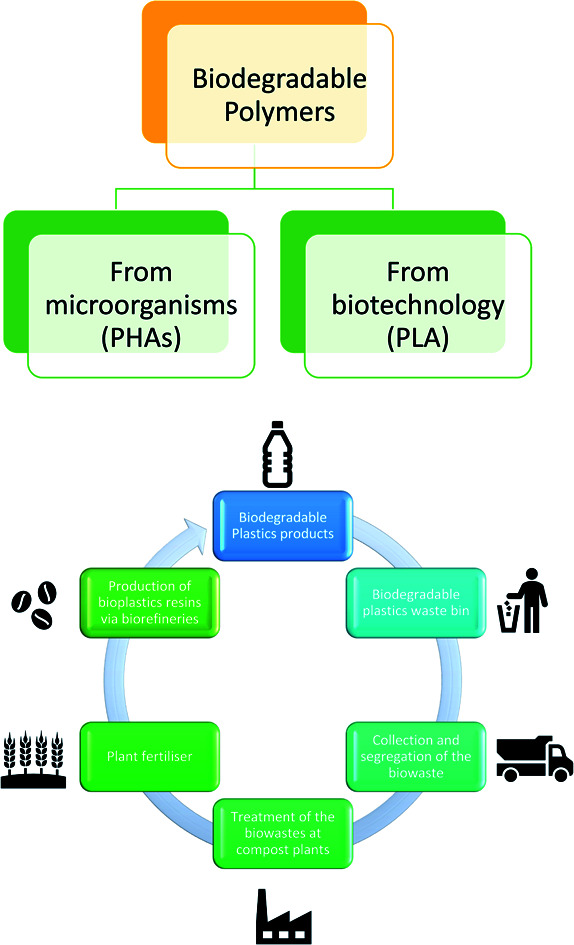

## 1. Introduction

**Fig. 1:**
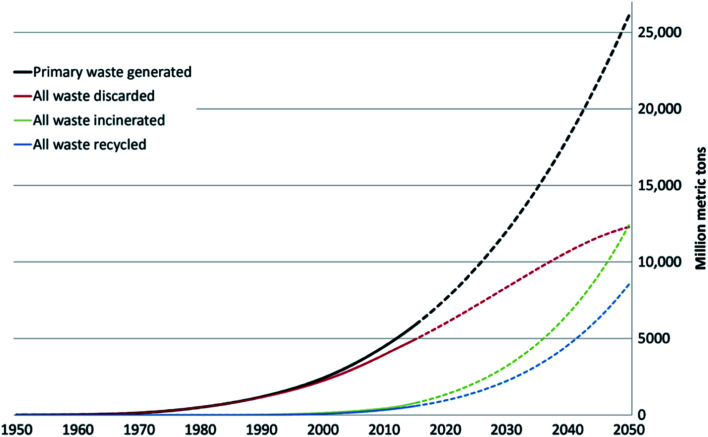
Cumulative plastic waste generation and disposal-solid lines show historical data from 1950–2010; dashed lined show projections of historical trends to 2050.^1^

**Fig. 2:**
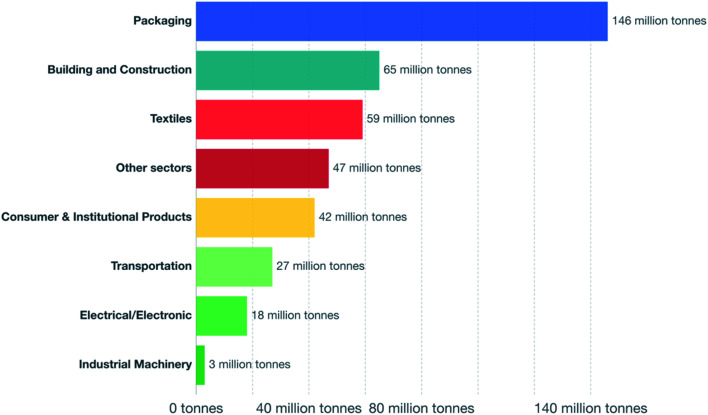
Primary plastic production by industrial sector, 2015.^1^

**Fig. 3:**
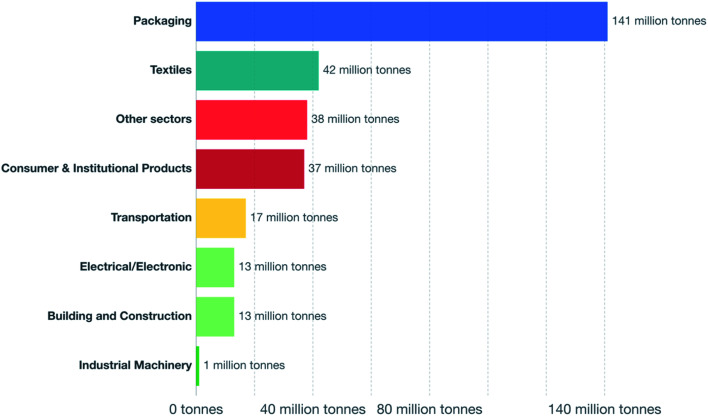
Plastic waste generation by industrial sector, 2015.^1^

The worldwide production of polymers has been continuously rising from 2 million tons in 1950 to around 381 million tons in 2015. This is approximately equal to the mass of two thirds of the global population and need to be disposed of by the end of their life cycle.^1,2^ The rapid production growth of plastics is extraordinary, surpassing most other man-made materials with the exception of steel and cement.^1^ In 2010, the plastic waste generation was estimated to be around 274 million tons. Fig. 1 represents the cumulative plastic waste generation and disposal between 1950 and 2010. Between the period of 1950 and 2015, the cumulative waste generation of primary (plastics manufactured from virgin materials) and recycled plastics was around 6300 million tons. Roughly, 800 million tons (around 12%) of plastics have been incinerated. 600 million tons (around 9%) have been recycled, and only 10% of which have been recycled more than once. Approximately, around 60% of all plastics ever produced until 2010 were discarded and are accumulating in the natural environment and landfills.^1^ To date, plastics do not experience significant recycling rates and hence are discarded or incinerated together with other solid waste.^3^ Between 22% and 43% of polymers end up in landfills, therefore, wasting the carbo feedstock and eventually leading to ground water pollution by the leaching of toxic additives. Waste from polymers accumulates in the natural environment in which they can remain for up to two thousand years.^4^ The pollution resulting from polymers is especially harmful in the marine environments, where 100 million tons of polymers can cause an ecosystem service damage of roughly US $ 13 billion per year.^5^ As per Fig. 2 and 3, packaging was the dominant use of primary plastics, with around 42% of plastics entering the use phase and it was also the dominant source for plastic waste with around 141 million tons. Fossil fuels trigger environmental concerns. The scientific evidence that carbon dioxide is one of the main reasons behind global warming and climate change is overwhelming. The CO_2_ continuous measurements at Mauna Loa since 1958 have conclusively shown increasing CO_2_ levels in more recent times. During this period, the data shows an increase at a rate of 1.5 ppm per year for the ambient concentration of CO_2_. A trend which if to continue the same, will mean doubling the CO_2_ levels by 2150.^6^ In the past 800 years, the average concentration of carbon dioxide in the atmosphere was estimated to be around 280 ppm. In 2019, the average concentration of CO_2_ increased to over 400 ppm. The critical limit of global warming has been adjusted by the United Nations from 2.0 °C to 1.5 °C; to prevent dramatic and irreversible changes in weather. These limits are expected to be surpassed in 20–40 years if no serious mitigations are taken.^7^ The dwindling nature, high price of petroleum, concerns about climate change, as well as the continued population growth are some of the factors that are urging the plastics industries to adapt sustainable natural solutions. Today, the worldwide population exceeds 7 billion people and is expected to reach to around 9 billion by 2050. Therefore, energy needs are also expected to increase. It is expected that there will be an increase in the electricity generation from 20 × 10^15^ W h in 2010 to around 31.2 × 10^15^ W h in 2030. As shown in Table 1, in 2010 the world energy supply has been dominated by fossil fuel. However, combustion of fossil fuel is a main cause of air pollution.^8^ Consumption of fossil fuels increases hunger for energy and will eventually lead to higher greenhouse and CO_2_ emissions. A sudden collapse of the biosphere might be triggered due to the progressive global warming and continuous reckless depletion of natural resources.^9–13^ Recent government policies that are focused on conservation of energy, as well as CO_2_ and footprint reduction are also driving the research about polymers towards the use of renewable and sustainable biopolymers. For example, Canada has announced that it is going to ban single use plastics by the end of 2021 in order to reach to zero plastic wastes by 2030.^14^ Furthermore, concerns about preservation of natural resources and climate change are considered worldwide motivations for academic and industrial researchers to reduce the consumption and dependence on fossil fuel.^15^ It is widely recognized that in order to meet the continuously increasing needs of materials of a constantly growing world population while at the same time maintaining functioning ecosystems, societies are required to switch to plant-based resources which are renewable on a short time scale and whose consumption and conversion are green.^16^ The main aim is to use biopolymers that contain the highest possible number of renewable resources to have a green future.^17,18^ Therefore, bioplastics are moving towards becoming the favorable option to be utilized in polymer manufacturing and various applications.^13,19–23^

**Table 1:** Total global energy consumption for 2010^8^

Types of energy	Energy consumption, %
Oil	35.3
Coal	27
Natural gas	20.5
Nuclear	5.0
Hydroelectric	5.8
Biomass	6.3
Other renewable	1.1

**Fig. 4:**
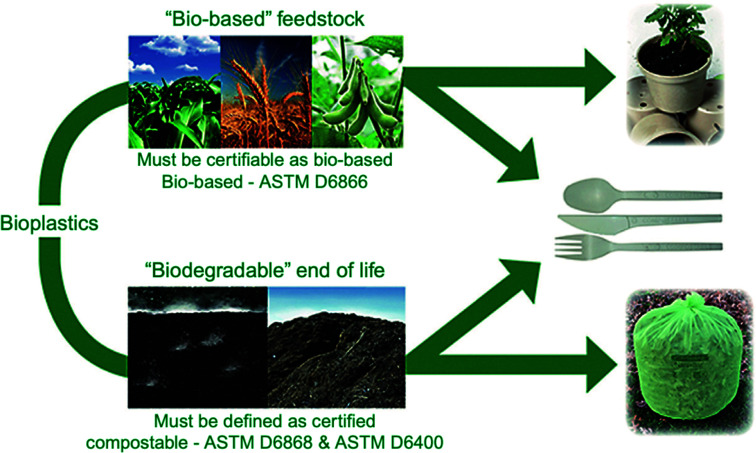
Difference between bio based and biodegradable bioplastics.^24^

**Fig. 5:**
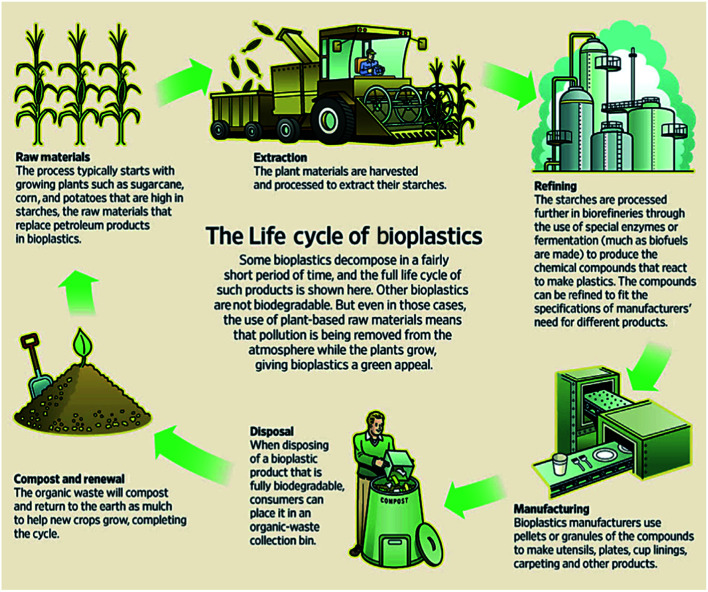
Life cycle of bioplastics.^24^

**Fig. 6:**
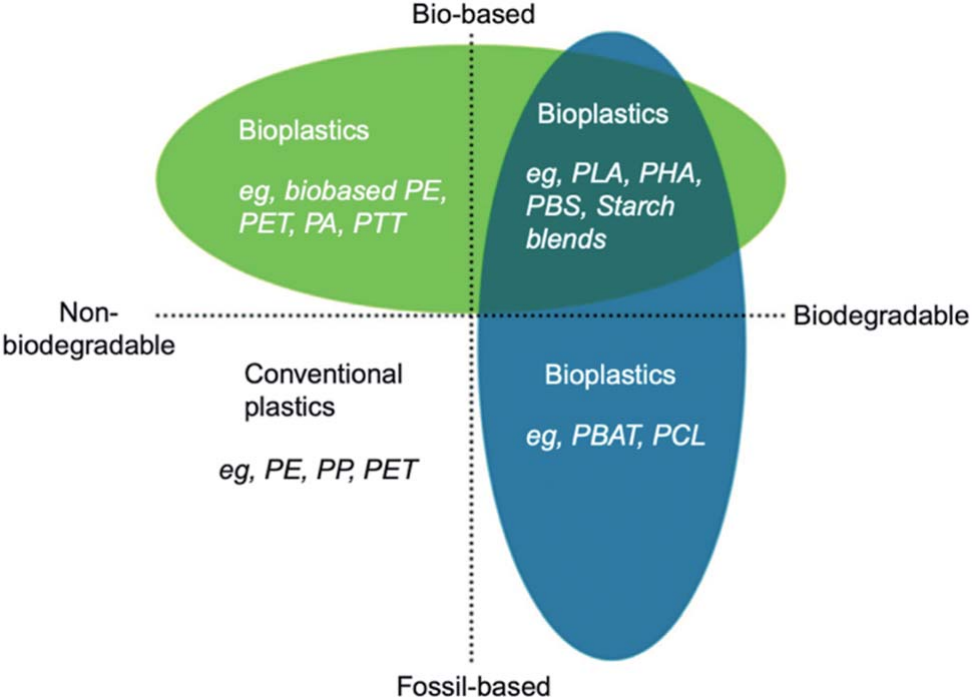
Materials' classification based on their biodegradability and bio-based content.^25^

**Fig. 7:**
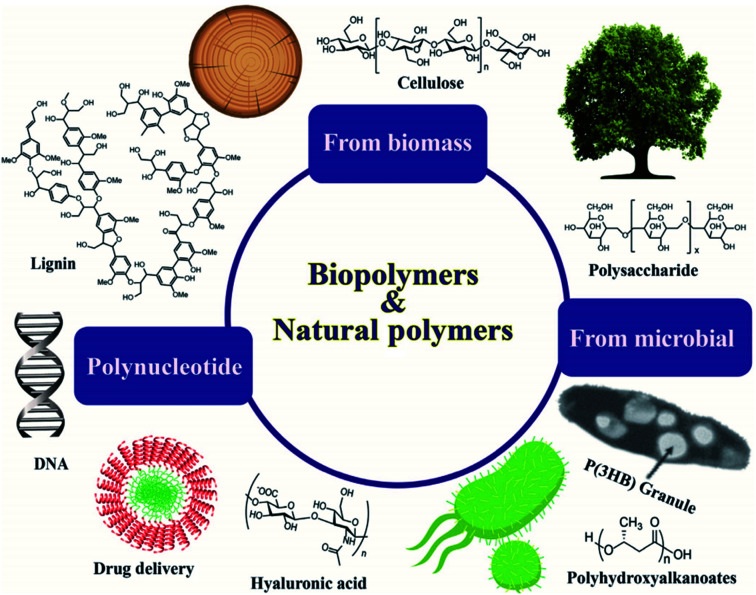
Classifications of biopolymers based on their origins.^26^

Bioplastics are one type of plastic which can be generated from natural resources such as starches and vegetable oils. Bioplastics are basically classified as bio based and/or biodegradable. Not all bio-based plastics are biodegradable and similarly not all biodegradable plastics are bio based. Bioplastics are referred to as bio based when the focus of the material is on the origin of the carbon building block and not by where it ends up at the end of its cycle life. Bio plastics are said to be biodegradable if they are broken down with the effect of the right environmental conditions and microbes which in turn use them as a food source. The bioplastics are considered compostable if within 180 days, a complete microbial assimilation of the fragmented food source takes place in a compost environment. The difference between the two branches of bioplastics is shown in Fig. 4.^24^ An illustration of the bioplastics' life cycle is presented in Fig. 5. The cycle initiates by growing plants that are rich in starches such as corn and sugarcane. The next step is the extraction of starches out of these plants by processing and harvesting the plants. The extracted starches are then refined and fermented by special enzymes to produce chemical compounds that produce plastics after they react. Plastics in the form of pellets are then used to manufacture products. After their full use, the manufactured products are then placed in an organic waste container and finally the last stage of the cycle begins.^24^Fig. 6 shows a classification of materials based on their biodegradability and bio-based content.^25^ As shown in Fig. 7, biopolymers can be classified according to their origin into three categories. Firstly, polymers that are made from renewable resource/biomass and agricultural resources. The second group contains polymers obtained from animal origin or microbial products and that are useful in pharmaceutical as well as medical applications. The last group includes chemically synthesized biodegradable polymers that have been altered from natural polymers or obtained from petrochemical resources.^26^

**Fig. 8:**
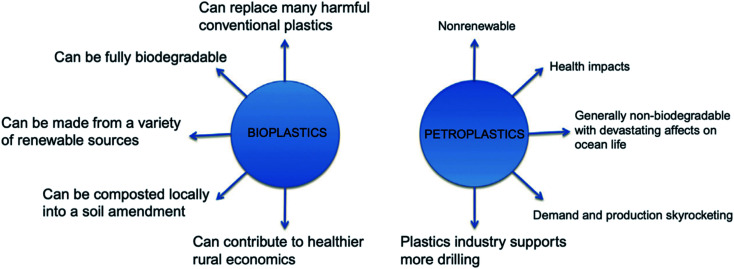
Comparison between bioplastics and petro plastics.^24^

**Fig. 9:**
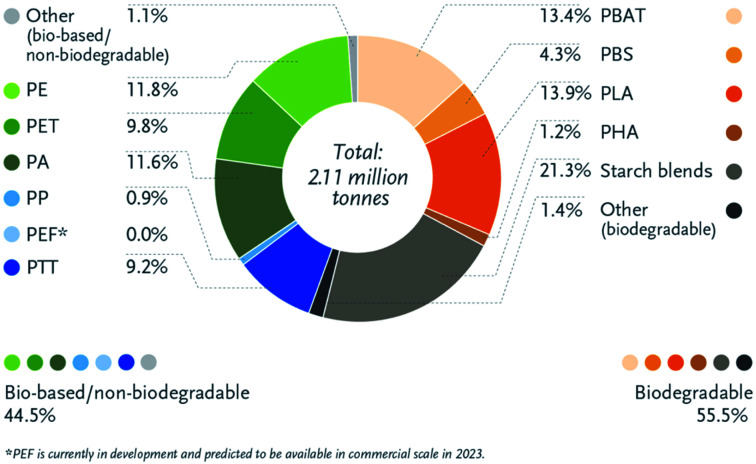
Global production capacities of bioplastics by material type, 2019.^27^

**Fig. 10:**
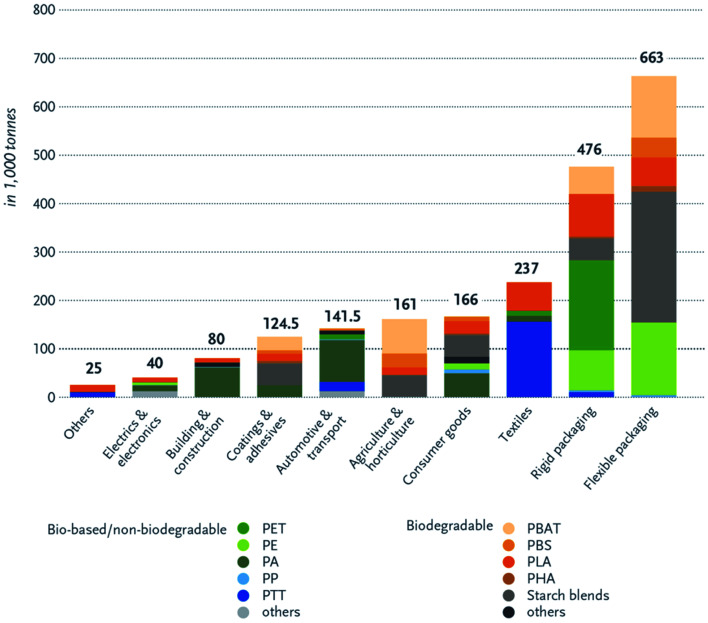
Global production capacities of bioplastics by market segment, 2019.^27^

**Fig. 11:**
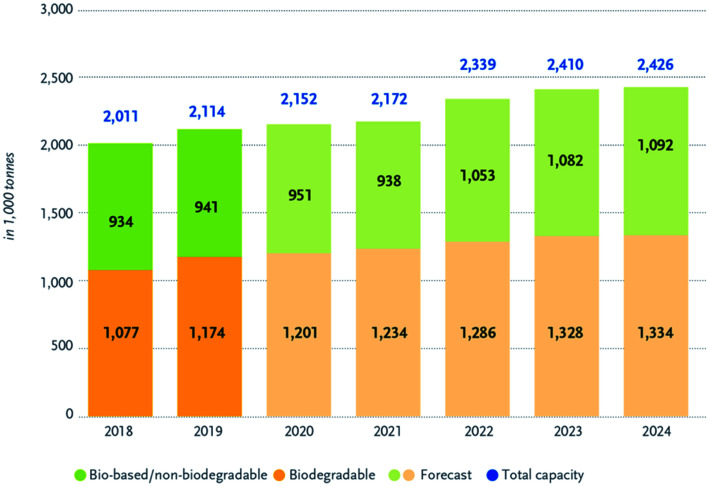
Global production capacities of bioplastics.^27^

Bioplastics have many advantages over petroleum-based plastics. The incorporation of bioplastics in different applications is under growth and development. Fig. 8 presents the main differences between bioplastics and petroleum based plastics.^24^ Bioplastic fast market growth is more than 8–10% per year. The global demand for bioplastics is expected to increase due to the availability of raw material, their renewability, their technical properties and advanced functionalities, their recycling solution, as well as their wide processing window (as shown in Table 2). Fig. 9 and 10 introduce the global production capacities of bioplastics in 2019 by material type, and market segment respectively. Fig. 11 shows global production of bioplastics between 2018 to 2024. Innovative and new biopolymers such as PHAs and polypropylene (PP) show the highest relative growth rate. All the bio-degradable plastics including PLA and PHAs accounts for over 1 million tons of the worldwide production capacities of bioplastics. The production of biodegradable plastics is estimated to increase to 1.33 million in 2024, this will mainly be due to PHA's vital growth rate. Bioplastics continued to be used in number of applications including but not limited to, packaging, electronics, catering, and automotive. With around 1.14 million tons (more than 53%), packaging continues to be the largest field for bioplastic applications in 2019. Nonetheless, applications such as building construction and automotive have increased their bioplastics share in a significant way. Based on the market data compiled by European Bioplastics in association with the research institute Nova-Institute, the global production of bioplastics is expected to raise to around 2.43 million tons in 2024 compared to around 2.11 million tons in 2019.^27^

**Table 2:** Processibility window for some typical commercial biodegradable polymers^28^^a^

Polymer	Injection molding	Extrusion	Extrusion blow molding	Cast film extrusion	Blow molding	Fiber spinning	Thermoforming
PLA	✓	✓		✓	✓	✓	✓
PHB	✓	✓	✓	✓	✓		✓
PHB–PHV	✓	✓	✓	✓	✓	✓	✓
PBS	✓	✓					
PCL	✓	✓	✓		✓	✓	✓
PBST	✓	✓		✓			✓
PBAT		✓	✓	✓			
PTMAT		✓	✓	✓		✓	
PVA	✓	✓		✓		✓	✓
PP, PE with additives	✓	✓	✓	✓	✓	✓	✓
Starch	✓	✓	✓	✓			
Starch with PVA	✓	✓		✓	✓	✓	
Cellulose	✓	✓			✓		
Starch with cellulose acetate	✓	✓	✓		✓		✓

a Abbreviations: PLA, poly(lactic acid); PHB, polyhydroxybutyrate; PHV, poly(hydroxyl valerate); PBS, poly(butylenes succinate); PCL, poly(ε-caprolactone); PBST, poly(butylene succinate terephthalate); PBAT, poly(butylene adipate terephthalate); PTMAT, poly(tetramethylene adipate terephthalate); PVA, poly(vinyl alcohol); PP, poly(propylene); PE, poly(ethylene); PVA, poly(vinyl alcohol).

**Fig. 12:**
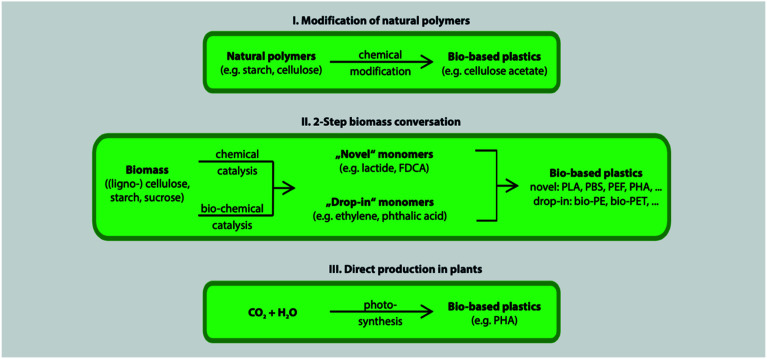
The three main approaches to bio-based plastics.^29^

**Fig. 13:**
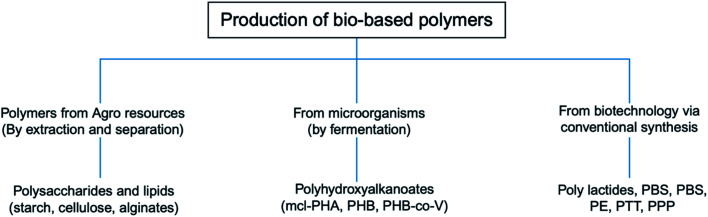
The three main ways for the production of bio-based polymers.^30,31^

**Fig. 14:**
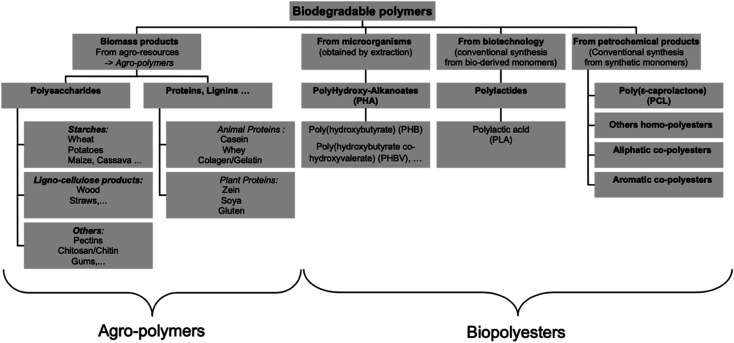
Main biodegradable polymers classification.^30^

Fig. 12 shows the three main routes to obtain bio-based plastics. The first approach is by alteration of natural polymers whilst preserving the polymer backbone intact. This method is used for the production of cellulose-based and starch plastics and it is considered today as the most important method. Furthermore, this approach is used for bio-based polymers and fibers that are used in non-plastic and non-food applications. The second approach consists of two steps biomass conversion that are normally complex and divided into various sub steps. The first step consists of the production of bio-based monomers *via* means of biochemical and/or chemical transformation. This step is usually followed by polymerization of the monomers in the final step. This approach is gaining much attention as a result of the advancements in the bio-technological and chemical production of monomers. Bio-based monomers that have not been applied to the market in the past or have new structures are called novel monomers. They are intended to replace standard plastics due to their enhanced functionality and hence additional markets and applications. Novel bio-based plastics require the development of new recycling systems. On the other hand, drop-in monomers are bio-based versions of conventional monomers. The use of drop-in monomers in the manufacturing of conventional plastics is beneficial. This is because they cannot be distinguished by performance nor structure from their petrochemical counterparts. This allows them to enter existing processing and recycling systems easily. The third approach includes the production of polymeric material directly inside microorganisms or plants without additional modification. This approach is becoming more feasible now, thanks to the progress in genetic engineering and biotechnology that made it possible to move genes responsible for polymer's production such as PHAs from bacteria into crops. This approach has been subjected to several studies, yet, no significant amount of bio-based plastics has been produced accordingly.^29^ In general, the production of bio-based polymers from renewable resources can be done in different ways as shown in Fig. 13 and 14. Firstly, by the use of natural bio-based polymers with partial modification in order to meet the requirements. A clear example of that would be starch. Secondly, the production of bio-based polymers from microbial production such as PHA. Thirdly, producing bio-based monomers by fermentation and conventional chemistry followed by polymerization such as PLA. Finally, polymers that are prepared from petrochemical products (synthetic monomers, such as polycaprolactone (PCL)).^30–32^ An overview of the commercially produced biodegradable polymers are shown in Table 3.^24^

**Table 3:** A list of the main biodegradable polymers and their suppliers (past & present)^24^^a^

Polymer	Trade name	Company
Starch	Solanyl	Rodenburg, Netherlands
PLA	NatureWorks PLA	Cargill, USA
	PLA	Galactic, Belgium
	l-PLA	Purac, Netherlands
	PLA-based resins: Bio-Flex and Biograde PLA	FKuR, Germany
	Lacty	Schimadzu, Japan
PHA	Nodax	Procter and Gamble, USA (previously), Danimer Scientific (present)
	Mirel	Metabolix, USA (discontinued)
	Biomer	Biomer, Germany
	ENMAT	TianAn Biologic Materials, China
PCL	Tone	Dow Chemicals, USA
	CAPA	Perstorp, UK
	Celgreen	Daicel, Japan
PEA	BAK	Bayer, Germany
Aliphatic polyesters	PBS	BASF, Germany
	PBS	Mitsubishi Gas Chemical, Japan
	PBS	Showa Highpolymer, Japan
	PBS	Ire Chemicals, Korea
	PBS	Anqing Hexing Chemical Co., China
Aliphatic copolyesters	PBSA Bionolle	Showa Highpolymer, Japan
	EnPol, PBSA	Ire Chemicals, Korea
	PBSA	Kingfa, China
	PBSA	IPC-CAS, China
Aromatic copolyesters (PBAT)	Biomax	DuPont, USA
	Eastar Bio	Eastman Chemicals, USA
	Ecoflex	BASF, Germany
	MATER-BI	Novamont, Italy

a Abbreviations: PLA, poly(lactic acid); PHA, polyhydroxyalcanoates; PCL, poly(ε-caprolactone); PEA, poly(esteramide); PBAT, poly(butylene adipate-*co*-terephthalate).

Biobased plastics are intended to reduce carbon emissions due to the fact that bio-based raw materials absorbed CO_2_ from the atmosphere. At the same time, biodegradable plastics are under research and development in order to reduce the pollution caused by petroleum-based plastics. This is because they degrade much faster than other conventional plastics. PLA is both: biobased and biodegradable under industrial composting conditions (at a high temperature, around 58 °C). Because of its good mechanical properties, processability, renewability, and non-toxicity, PLA is considered today as one of the most commercially promising bioplastics. When compared with most other biodegradable polymers, PLA has better durability, transparency, and mechanical strength. PLA's global production volume was estimated to be around 190 000 tons in 2019 and is expected to double every 3–4 years. PLA has been used in single use applications and disposable packaging products such as food packaging.^7,33^

PHAs are a significant polymer family that are 100% bio-based and bio-degradable. PHAs are microbiologically produced polyesters that have tunable physical and mechanical properties. This is accompanied by low environmental impact due to their biodegradability and non-toxicity nature. Therefore, they are promising candidates for a sustainable future manufacturing. Ranging from brittle thermoplastics to gummy elastomers, PHAs' properties can be altered by the selection of bacteria, fermentation conditions, and substrate. Due to their flexible properties, PHAs can eventually substitute PP, polyethylene (PE), and polystyrene (PS), which are the main polymers of today's global polymer market.^34^ Biodegradable polymers such as PHAs have the potential to lower the amount of pollution caused by the constantly growing demand of polymers. Compared to PLA, PHAs are both compostable and biodegradable in marine environments. On the other hand, PLA is compostable but may stay for up to a thousand years in the marine environment.^4^ PHAs' biocompatibility is another important aspect. Due to the facts the PHAs are non-toxic, and they occur naturally in human tissues and blood, they have been used in medical applications.^35^ A drop of fossil energy use by 95% and greenhouse gas emission by 200% can be achieved by substituting petroleum-based polymers with PHAs.^4^ Therefore, PHAs have the potential to contribute to a green industrial evolution.^36,37^

The objective of this work is to focus on both PLA and PHAs as alternative, affordable, sustainable, biodegradable materials that can replace petroleum-based polymers in a wide range of industrial applications. Therefore, physical, thermal, rheological, and mechanical properties of both polymers as well as their permeability and migration properties have been reviewed. Furthermore, PLA's recyclability, sustainability, and environmental assessment have been also reviewed. The combination of all of these aspects for both of PLA and PHAs polymers in literature is rare. The main aim of this review is to gain a better understanding of the role of these aspects for the purpose of widening the usage of both of these polymers in different applications and therefore contribute to lowering both, the amount of waste resulting from petroleum-based plastics and their pollution in the environment.

## 2. Poly(lactic acid)

**Fig. 15:**
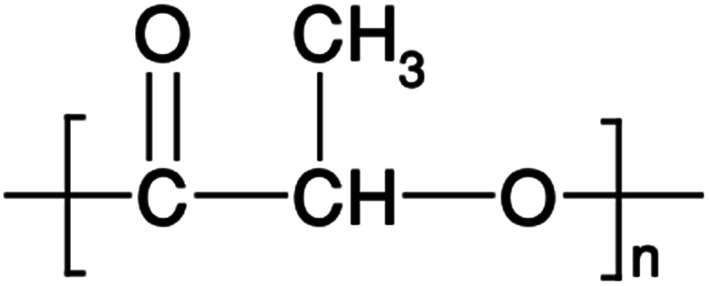
The chemical structure of PLA.^30^

**Fig. 16:**
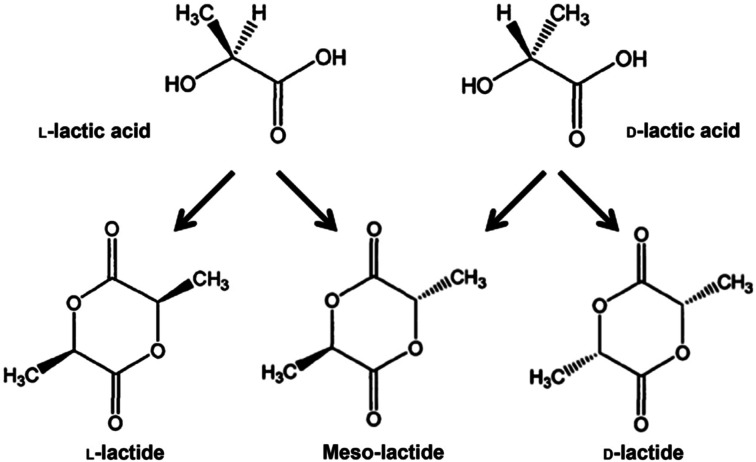
The optical monomers of lactic acid along with the three stereo form of lactides.^38^

**Fig. 17:**
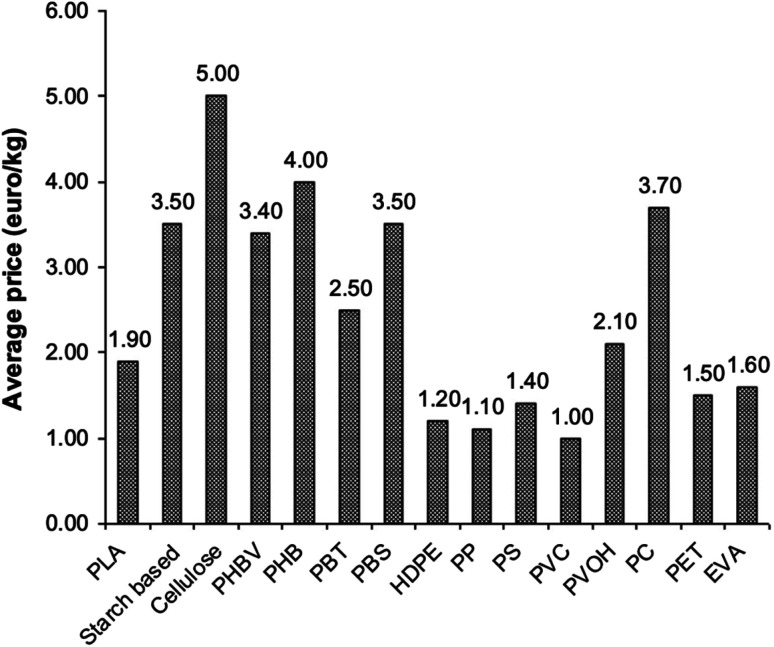
Average prices of various polymers in 2009.^49^

**Fig. 18:**
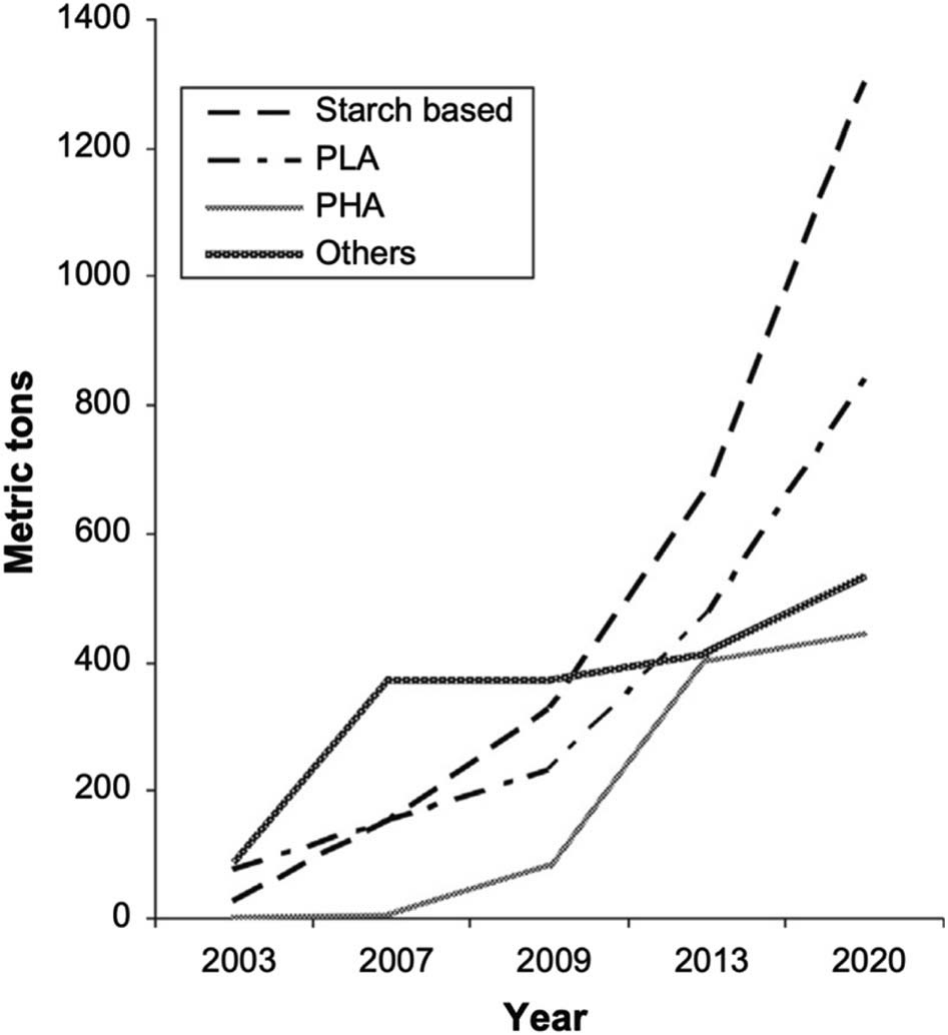
Renewable biodegradable polymer's global production in 2003 to the projection for 2020.^49^

PLA's chemical structure is shown in Fig. 15. The raw material used in the synthesis of PLA is the high purity monomer, lactide. Lactide can be obtained in two synthesis steps: oligomerization of lactic acid (LA) followed by cyclisation. Lactic acid optical monomers consist of l-lactic acid and d-lactic acid. From both optical monomers, three possible stereo forms of lactide can be formed from the oligomer of lactic acid as shown in Fig. 16. These stereo forms are: ll-lactide (LLA), dd-lactide (DLA) and dl-lactide (meso-lactide (MLA)).^38^ LA is the basic building block for the production of PLA. LA is chemically known as 2-hydroxy-propionic acid with chiral stereoisomers l (−) and d (+). Naturally occurring LA is mainly found in the l form, while chemically synthesized LA can be a racemic d and l mixture. LA is a highly water-soluble and is a biologically stable substance. PLA is a rigid thermoplastic polymer that is classified under the family of aliphatic polyesters. PLA is mainly derived from renewable resources, particularly sugar and starch. The PLA family includes poly(l-lactide) (PLLA), poly(d-lactide) (PDLA), poly(dl-lactide) (PDLLA), poly(meso-lactide), and copolymers obtained from the monomers.^39^ Polymerization of l-lactide yields poly(l-lactide) while poly(d-lactide) is produced by polymerization of d-lactide. Based on the stereochemistry of the polymer backbone, PLA can be semicrystalline or amorphous. PLLA and PDLA are semi-crystalline, while PDLLA and poly(meso-lactide) are amorphous.^30,39–42^ Due to its relatively low price and availability, PLA is considered to be one of the highest potential bio polyesters for packaging and medical applications. Today, different companies produce a wide range of PLA products with various l/d ratios.^43–47^ The enantiomeric purity of lactic acid stereo-copolymers affects the physical properties of polylactide. PLA's wide availability and tunability made it a strong alternative to conventional plastics for packaging applications such as: cups, bottles and trays.^44,46,47^ Reports in literature confirm that PLA's biodegradation does not result in any eco-toxicological effect.^48^Fig. 17 gives an overview about the prices of both, conventional and biodegradable plastics in 2009. It is clear that PLA's price is the lowest of all biodegradable polymers. In addition, as the figure suggests, the nearest competitor to conventional polymers such as: PE, PP, PS, polyethylene terephthalate (PET), and Ethylene Vinyl Acetate (EVA) is PLA. At the same time, there is a great potential for PLA to replace polycarbonates (PC), this is because when compared to PC, the price of PLA is significantly lower. PLA can replace PC in various applications, specifically, in electronics'/electric's casings.^49^Fig. 18 shows that the future of PLA's production is promising, and it has the potential to overtake the sum of other biodegradable polymers such as Poly(Butylene Succinate) (PBS) and PCL.

### 2.1 PLA's synthesis

**Fig. 19:**
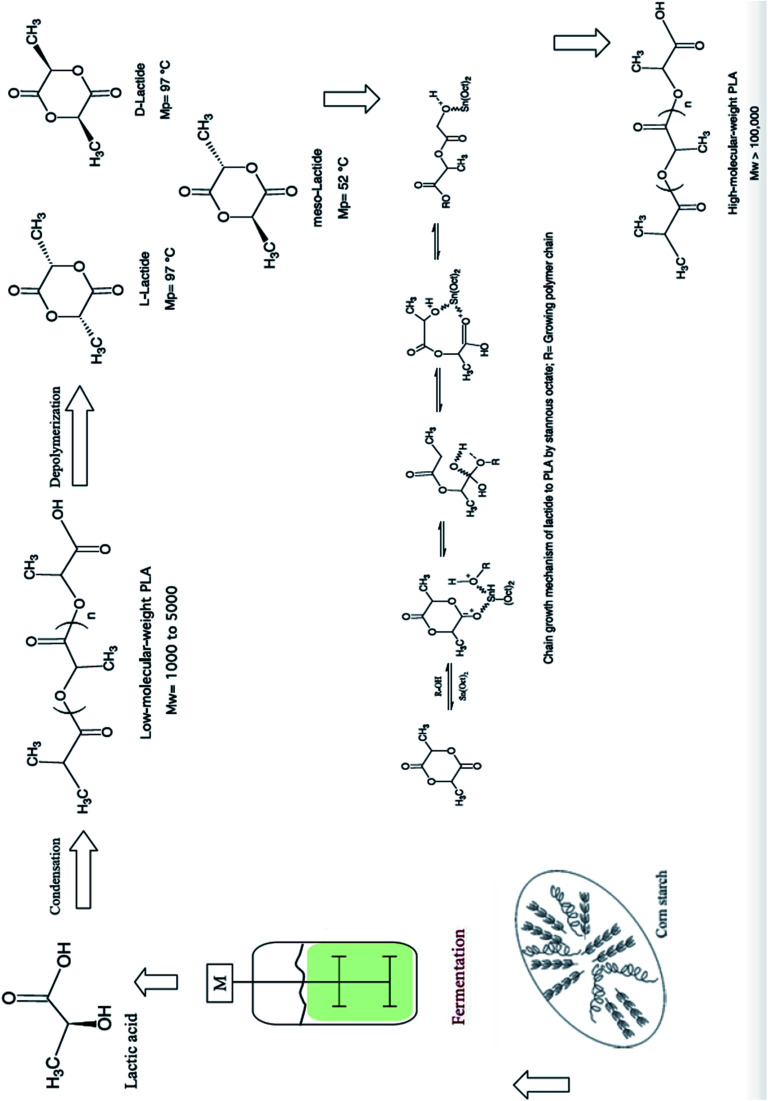
PLA's production steps.^28,55^

**Fig. 20:**
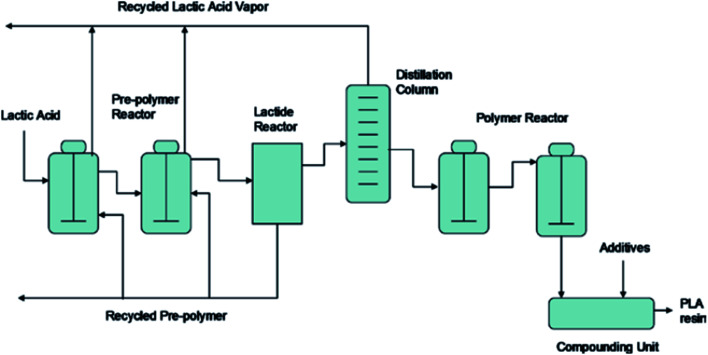
ROP process for PLA as reported by NatureWorks LLC.^59^

The synthesis for PLA begins with LA production, then lactide formation, and it finally ends with LA polymerization.^50^ The synthesis of PLA can be accomplished in three steps which are: firstly, the production of LA by microbial fermentation, secondly, the purification of LA followed by lactide preparation, and finally, polycondensation of LA or Ring Opening Polymerization (ROP) of lactides.^51^Fig. 19 shows PLA's production steps.^28^ Polycondensation is the cheapest route, and it includes both: solution and melt polycondensations. Yet, it is hard to produce a solvent-free PLA with high molecular weight using these routes.^46^ This can be attributed to the extended reaction times and the use of solvent in direct condensation which results in low to intermediate molecular weights. The production of high molecular weight PLA was found to be possible by polycondensation through the use of chain extension.^52^ Defined as bifunctional groups with low molecular weight compounds, chain extenders increase biopolymers' molecular weights in a rapid reaction.^53^ High molecular weight polymers can be achieved by azeotropic condensation polymerization without using chain extenders or adjuvants. However, high amount of catalyst impurities is expected as a result of the high levels required for acceptable reaction rates. Problems such as catalyst toxicity, un-desirable degradation, and un-controlled hydrolysis rates can be resulted from residual catalysts. The addition of phosphoric or pyrophosphoric acid can deactivate the catalyst. It is recommended to use two equivalents of acid to divalent tin catalyst. This can assist in enhancing polymers' heat and storage stability as well as the weathering resistance. The addition of strong acids such as sulfuric acid can be used to precipitate and filter the catalyst.^54^ However, the most common route to produce high molecular weight PLA is through ROP. The purified LLA, DLA, and MLA are converted into corresponding high molecular weight polyester *via* catalytic ROP.^50^ ROP process involves the ring opening lactide in the presence of catalyst. The process includes three main steps, which are: polycondensation, depolymerization and ROP.^55,56^ This route involves extra purification steps which are relatively expensive and complicated. Controlling both: the ratio and sequence of d- and l-LA units in the final polymer is feasible by controlling the residence time and temperatures along with the type of catalyst and its concentration.^57^ Transition metals such as lead, tin, zinc, yttrium, bismuth, and aluminium can be used as catalysts.^58^ PLA is chemically synthesized by heavy metal catalyst. Yet, for some applications such as biomedical and food applications, the trace residues of such heavy metal catalysts are undesirable. Therefore, replacing these heavy metal catalysts with safe and environmentally acceptable candidates is a high priority aim. Fig. 20 shows PLA's ROP process as reported by NatureWorks LLC.^59^

Due to its environmentally friendly nature as well as the fact that it can be carried out under mild conditions, enzymatic polymerization has been reported as one of the most feasible alternatives for synthesis of polymers.^60^ Synthesizing fine structure polymers from low-cost raw materials can be achieved with highly specific enzymatic reactions. On the other hand, chemical processes need elevated temperatures, extremely pure monomers, and anhydrous conditions to avoid side reactions.^61^ Therefore, enzymatic polymerization using LA-polymerizing enzyme can be the preferred PLA biosynthetic process to replace chemical synthesis methods. Yet, research is still going today to discover natural PLA producing microbes. Moreover, one of the most preferred methods is the one-step microbial production of PLA. This is because it is capable of controlling the composition of polymer *via* combining metabolic intermediates of LA monomers at different ratios in a single step process. One-step processes for the synthesis of PLA can be achieved utilizing PLA lactic acid bacteria strains.^62^

### 2.2 PLA's physical & thermal properties

PLA's physical, mechanical, and rheological properties are all affected by its glass transition temperature (*T*_g_).^63^ PLA can be manufactured into various useful items using thermal processes, such as extrusion and injection molding. As a result, PLA's rheological properties, especially its shear viscosity, have significant impact on the thermal processes. PLA's melt rheological properties have a significant impact on the polymer flow conditions during the processing stage. Generally, high molecular weight PLA has melt viscosities in the order of 500–1000 Pa at shear rates of 10–50 s^−1^. Such polymer grades are equivalent to molecular weight of 100 000 g mol^−1^ for injection molding to that of 300 000 g mol^−1^ for film cast extrusion applications.^54^ Low molecular weight PLA, that is roughly around 40 000 g mol^−1^ shows Newtonian-like behavior at shear rates for typical film extrusion, whereas a pseudoplastic, non-Newtonian fluid behavior has been reported for the melts of high molecular weight PLA. Compared to amorphous PLA, semi crystalline PLA tends to exhibit higher shear viscosity under similar processing conditions. Furthermore, when the shear rates increase, the viscosities of the melt decrease drastically, that is, the polymer melt shows a shear-thinning behavior. The glass transition temperature and the melting point (*T*_m_) are the two physical parameters required to predict the behavior of semicrystalline PLA. On the other hand, the glass transition temperature is the vital physical parameter in predicting amorphous PLA's behavior.^46,64,65^ Crystalline PLLA has been reported to have a density of 1.290 g ml^−1^, while amorphous PLLA's density was reported to be 1.248 g ml^−1^. PLA of 100% crystallinity has been reported to have a melt enthalpy of 93 J g^−1^. Yet, higher values of up to 148 J g^−1^ have been also reported.^66^Table 4 shows the physical properties of PLA along with other biopolymers.

**Table 4:** PLA's physical properties along with other biopolymers^67^^a^

Properties	PLA	PLLA	PDLLA	PGA	PDLLA/PGA (50/50)	PDLLA/PGA (75/25)	PCL	PHB
Density (g cm^−3^)	1.21–1.25	1.24–1.30	1.25–1.27	1.50–1.71	1.30–1.40	1.3	1.11–1.146	1.18–1.262
Tensile strength (MPa)	21–60	15.5–150	27.6–50	60–99.7	41.4–55.2	41.4–55.2	20.7–42	40
Young's modulus (GPa)	0.35–3.5	2.7–4.14	1–3.45	6.0–7.0	1–4.34	1.38–4.13	0.21–0.44	3.5–4
Elongation at break (%)	2.5–6	3.0–10.0	2.0–10.0	1.5–20	2.0–10.0	2.5–10	300–1000	5.0–8.0
Specific tensile strength (N m g^−1^)	16.8–48.0	40.0–66.8	22.1–39.4	40.0–45.1	30.9–41.2	31.8–42.5	18.6–36.7	32.0–33.9
Specific tensile modulus (kN m g^−1^)	0.28–2.80	2.23–3.85	0.80–2.36	5.00–4.51	0.77–2.14	1.06–2.12	0.19–0.38	2.80–2.97
Glass transition temperature (°C)	45–60	55–65	50–60	35–45	40–50	50–55	(−60)–(−65)	15.0–5.0
Melting temperature (°C)	150–162	170–200	Amorphous, no melt	220–233	Amorphous, no melt	Amorphous, no melt	58–65	168–182

a Abbreviations: PLA, poly(lactic acid); PLLA, poly(l-lactic acid); PDLLA, poly(d,l-lactic acid); PGA, poly(glycolide); PCL, poly(ε-caprolactone); PHB, polyhydroxybutyrate.

**Fig. 21:**
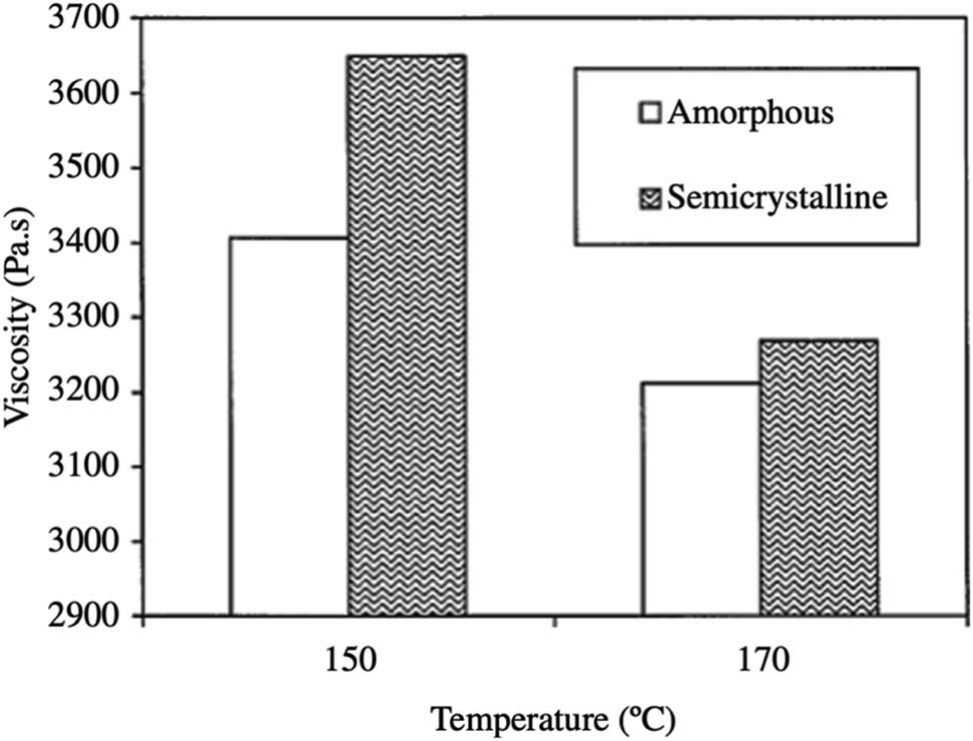
Melt viscosity of semi crystalline and amorphous PLA at different temperatures.^68^

**Fig. 22:**
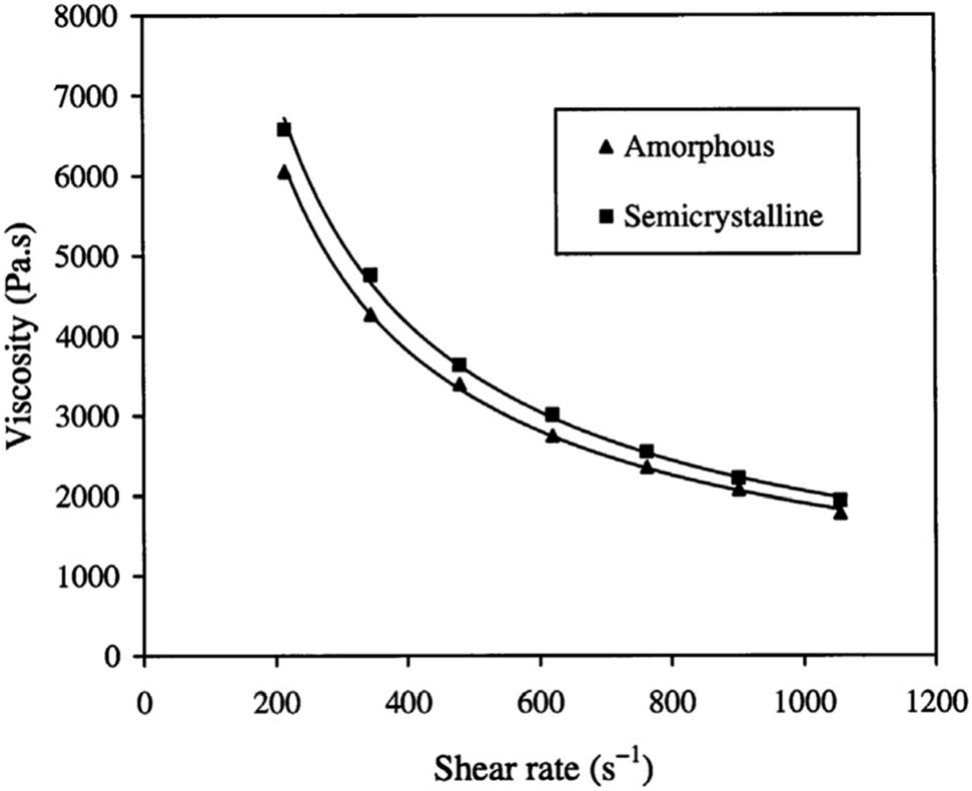
Shear rate impact on the melt viscosity for both semi crystalline and amorphous PLA.^68^

If the PLA contains higher than 93% of the l-lactic it is said to be semi-crystalline. On the other hand, PLA with lower optical purity-that is a PLA with 50–93% l-lactic is totally amorphous. Based on that, controlling the l/d ratio is crucial in deciding upon the crystallinity of polymer. The decrease of both: the extent and rate of PLLA crystallization is mainly due to macromolecular imperfections. Commercially, the majority of PLA is made from l- and d,l lactide copolymers; this is because PLA production often includes some meso-lactide impurities.^30^ In a study conducted by Fang and Hanna, the rheological properties of both semi crystalline and amorphous PLA resins have been obtained by attaching a tube rheometer to an extruder. The investigation was done at temperatures of 150 °C and 170 °C. Fig. 21 summarizes their results and shows that at higher temperatures, semi crystalline PLA exhibited a higher viscosity than amorphous PLA. This is attributed to the variation in the molecular structure. In the semi crystalline PLA, the molecules are arranged in an organized pattern. This has resulted in a relatively large flow resistance due to the stronger intermolecular forces. On the other hand, in amorphous PLA, the molecules are arranged in a random form which resulted in less flow resistance. Generally, semi crystalline structures offer stronger physical and mechanical properties than amorphous materials. A drop in the shear viscosities for both semi crystalline and amorphous PLA was observed when the temperature increased. Higher viscosity values were obtained at 150 °C when compared to those attained at 170 °C. This is due to the fact that at high temperatures, the connections between the molecular chains become weaker as a result of the higher vibrational amplitude of the PLA molecules, which transforms the melt to flow smoothly. In addition, the viscosity' of PLA melt is highly affected by the shear rate. As observed in Fig. 22, as the shear rate increases for both semi crystalline and amorphous PLA, values of the viscosity for both drop drastically. The relationship between the shear rate and the viscosity is nonlinear but shows a typical non-Newtonian pseudoplastic behavior. This can be explained by the strong shearing action during extrusion that resulted in breaking the molecular chains down.^68^

**Fig. 23:**
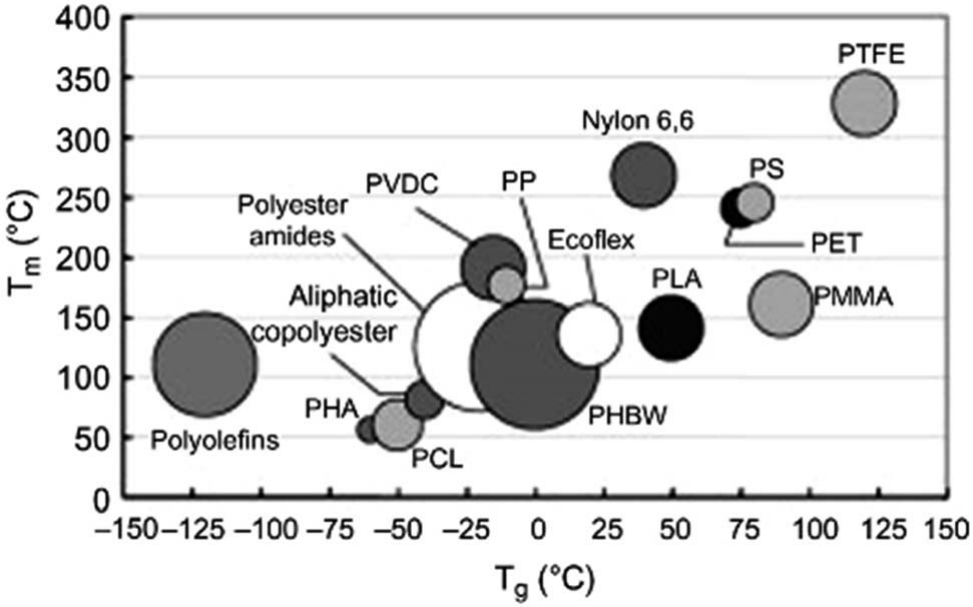
Melting and glass transition temperature of PLA and some other polymers.^39^

**Fig. 24:**
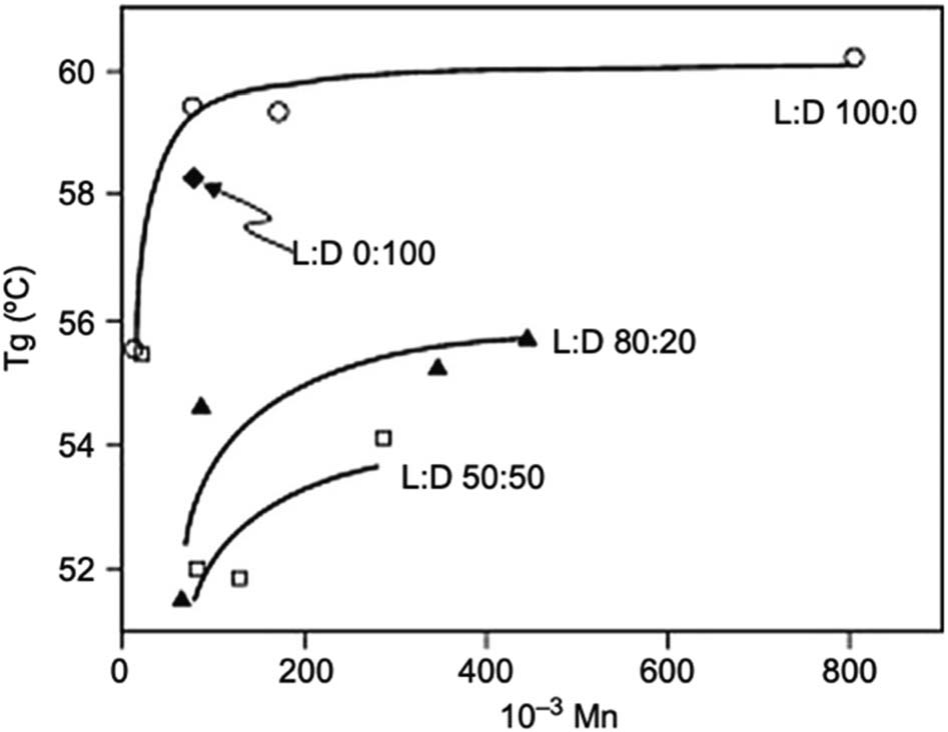
A comparison of glass transition temperature of PLAs with various l-lactide and d-lactide contents as a function of molecular weight.^73^

The thermal properties of PLA, its crystallinity degree as well as its crystallization depend on the polymer molecular weight, polymerization conditions, thermal history, and purity.^50^ Generally, *T*_g_ of polymers depends upon the number average molecular weight, microstructure, and stereocomplex configuration.^69^ Typically, *T*_g_ of PLA is in the range of 50 to 80 °C and its *T*_m_ is in the range of 130 to 180 °C. A pure PLA fully in l or d stereochemistry has a *T*_g_ of 60 °C, and a melting point of 180 °C.^70^Table 5 presents the glass transition and melting temperatures for PLA copolymers along with PET.^71^ PLA has relatively low melting temperature and high glass transition temperature when compared to other polymers. Fig. 23 shows a comparison of the melting temperature and glass transition temperature of PLA with other polymers.^39^ Achmad *et al.* have reported that both PLLA and PDLA are semicrystalline polymers with melting points of around 180 °C. At the same time, they have also reported that the copolymer PDLLA is an amorphous material with a glass transition temperature of only 50–57 °C.^72^ A comparison of glass transition temperature of PLA with various optical purity of the polymer with respect to their molecular weights is shown in Fig. 24. As shown, PLA's *T*_g_ is dependent on the optical purity and the molecular weight of the polymer. *T*_g_ for PLA increases with molecular weight. PLA with higher l-lactide content exhibits higher *T*_g_ values than the same polymer with the same content of d-lactide.^73,74^ As the l-content decreases, crystallinity, melting temperature, and glass transition temperature all decrease.^73,75^ In case of semi-crystalline PLA, the melting temperature is a function of the initial PLA structure as well as the various processing parameters. A presence of meso-lactide in the structure reduces the melting temperature. With increasing the molecular weight, the crystallinity decreases, however, the melting temperature increases until it approaches a maximum value.^76^ Furthermore, Table 6 shows how the molecular number (*M*_n_), *T*_g_, *T*_m_, enthalpy, and crystallization temperature (*T*_c_) of PLA can be different for various types of lactide isomers.^77^ It can be observed that irrespective of whether the isomer type is l or d, both *T*_g_ and *T*_m_ increase with an increase in *M*_n_.

**Table 5:** Glass transition and melting temperatures for PET and a range of PLA copolymers^71^^b^

Copolymer ratio	Glass transition temperature (°C)	Melting temperature (°C)
PET	80	255
100/00 (l-/d,l) PLA	63	178
95/05 (l-/d,l) PLA	59	164
90/10 (l-/d,l) PLA	56	150
85/15 (l-/d,l) PLA	56	140
80/20 (l-/d,l) PLA	56	(125)^a^

a Melting point achieved by strain crystallization.

b Abbreviations: PET, poly(ethylene terephthalate); PLA, poly(lactic acid).

**Table 6:** The effect of isomers type on PLA's thermal properties^77^

Type of isomer	*M*_n_ × 10^3^	*M*_w_/*M*_n_	Glass transition temperature (°C)	Melting temperature (°C)	Melting enthalpy (J g^−1^)	Crystallization temperature (°C)	Crystallization enthalpy (J g^−1^)
l	4.7	1.09	45.6	157.8	55.5	98.3	47.8
dl	4.3	1.90	44.7	—	—	—	—
l	7.0	1.09	67.9	159.9	58.8	108.3	48.3
dl	7.3	1.16	44.1	—	—	—	—
d	13.8	1.19	65.7	170.3	67.0	107.6	52.4
l	14.0	1.12	66.8	173.3	61.0	110.3	48.1
d	16.5	1.20	69.1	173.5	64.6	109.0	51.6
l	16.8	1.32	58.6	173.4	61.4	105.0	38.1

### 2.3 PLA's mechanical properties

Depending on various parameters such as: polymer structure, material formulation (blends, plasticizers, composites, *etc.*), orientation, crystallinity, and molecular weight, the mechanical properties of commercial PLA can be diverse, ranging from elastic soft to stiff, high-strength materials. Developed by NatureWorks LLC, a summary of PLA's mechanical properties along with some physical and thermal properties is shown in Table 7.^78^ Similar to PS, PLA is a brittle material with low elongation at break and impact strength. Nonetheless, it is comparable to PET when it comes to its tensile strength and modulus. A comparison between the mechanical properties of PLLA, PS and PET is shown in Table 8.^79^ Due to its poor toughness, the use of PLA in applications that requires plastic deformation at higher stress levels has been avoided. This has opened the door to develop various modification techniques to improve the mechanical properties of PLA, specifically its toughness. Such techniques involve, blending with other polymers, the use of plasticizers, the addition of reinforcing fillers and fibers as well as nucleating agents.^80^

**Table 7:** A summary of PLA's mechanical properties along with some physical and thermal properties as reported from NatureWorks LLC^78^^a^

Properties/applications	Ingeo™ 2003D	Ingeo™ 3052D	Ingeo™ 3801X	ASTM method
Specific gravity	1.24	1.24	1.25	D792
Melt flow rate, g per 10 min (210 °C, 2.16 kg)	6	14	8	D1238
Relative viscosity	NP	3.3	3.1	—
Clarity	Transparent	Transparent	Opaque	—
Tensile strength at break, psi (MPa)	7700 (53)	NP	NP	D882
Tensile yield strength, psi (MPa)	8700 (60)	9000 (62)	3750 (25.9)	D882
Tensile modulus, kpsi (GPa)	500 (3.5)	NP	432 (2.98)	D882
Flexural strength, psi (MPa)	NP	15 700 (108)	6400 (44)	D790
Flexural modulus, psi (MPa)	NP	515 000 (3600)	413 000 (2850)	D790
Tensile elongation (%)	6.0	3.5	8.1	D882
Notched Izod impact, ft-lb per in (J m^−1^)	0.3 (16)	0.3 (16)	2.7 (144)	D256
Heat distortion temperature (°C)	55	55	65 (at 66 psi)	E2092
			140 (at 16.5 psi)	
Melt temperature (°C)	210	200	188	—
Crystallinity melt temperature (°C)	NP	145–160	155–170	D3418
Glass transition temperature (°C)	NP	55–60	45	D3418
Applications	Designed for fresh food packaging and food service ware applications such as: dairy containers, food service ware, transparent food containers, hinged ware, cold drink cups	Designed for injection molding applications that require clarity with heat deflection temperatures lower than 49 °C. Applications include: cutlery, cups, plates and saucers, as well as outdoor novelties	Designed for non-food contact injection molding applications that require opaque molded parts with heat deflection temperatures between 65 °C and 140 °C	—

a NP: not provided.

**Table 8:** A comparison of the mechanical properties of PLLA, PS and PET^79^^a^

Polymer	Tensile strength (MPa)	Tensile modulus (GPa)	Percentage elongation	Notched Izod (J m^−1^)
PLLA	59	3.8	4–7	26
PS	45	3.2	3	21
PET	57	2.8–4.1	300	59

a Abbreviations: PLLA, poly(l-lactic acid); PS, poly(styrene); PET, poly(ethylene terephthalate).

### 2.4 PLA's permeability

**Fig. 25:**
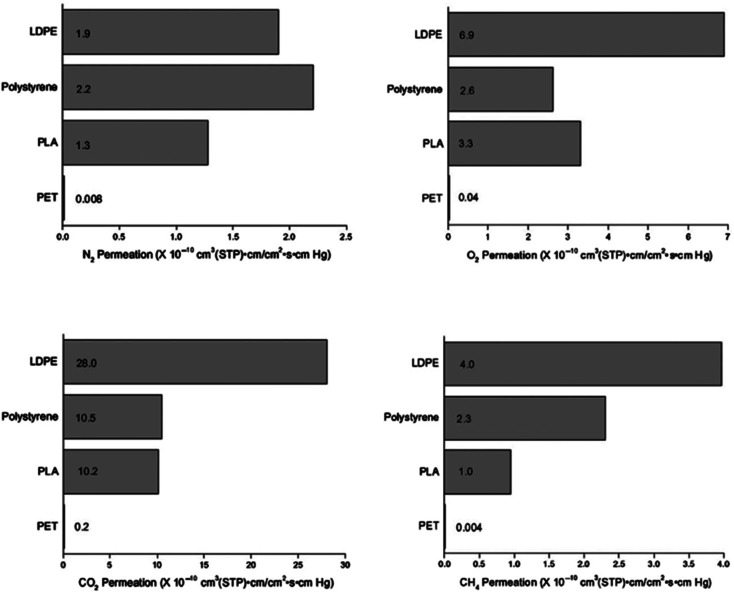
Properties of permeation for 100% linear PLA with an l : d ratio 96 : 04 compared to other plastics commonly used for packaging at 30 °C.^81^

When considering PLA as a packaging material, PLA's gas permeation properties are of significant importance. Packaging requires materials that have low-permeability, in order to prevent the occurrence of oxidation and the loss of flavor and aroma which may reduce the shelf-life of food. Since PLA is a biodegradable polymer with the potential to replace conventional petroleum based plastics, it crucial for PLA to have as effective permeability properties as these existing polymers.^33^ A study on gas permeation of PLA for oxygen, methane, nitrogen, and carbon dioxide was conducted by Lehermeier *et al.*^81^ The study concluded that PET's permeability was lower than that for PLA, which means that PET has superior barrier properties to PLA with an l : d ratio of 96 : 4. This is because PET contains aromatic rings in the polymer chain backbone, which decreases free volume and chain mobility. Crystallization can substantially improve the barrier properties. The increase of crystallinity in biaxially orientated PLA film with an l : d ratio of 95 : 5 with 16% crystallinity resulted in a drop of the permeability to 4.5 times lower than PLA film samples that have l : d ratio of 96 : 4 and 98 : 2 with 1.5% and 3% crystallinity, respectively. This is because crystallinity enhances the compactness of the structure, which makes it difficult for gas molecules to diffuse through the film. Fig. 25 presents a comparison of the permeation properties of 100% linear PLA with an l : d ratio of 96 : 04 with other polymers that are mainly used for packaging. In general, compared to both PS and LDPE, PLA possesses better barrier properties. With respect to nitrogen, carbon dioxide, and methane, PLA exhibits good barrier properties. On the other hand, PLA demonstrates a slightly weaker barrier properties for oxygen. This finding is significant; because it shows that PLA can be utilized as a robust packaging material to replace different petroleum-based plastic films. PLA's biodegradability, its “green” production along with its good barrier properties have made PLA a strong future packaging material candidate.^81^ For packaging materials, permeability to water is also of great importance. The water vapor permeability of different biodegradable polymers was studied and compared by Shogren.^82^Table 9 shows the water transmission rates for these materials. In comparison to many biodegradable polymers, PLA exhibits good water resistance except when it is compared to PHBV. Annealing of PLA at 130 °C prompts the formation of a crystalline structure, which in turns enhances water resistivity. This is because the molecular cross-sectional area for diffusion decreases while the diffusion path length increases due to crystallization, imposing restraints on the mobility of the amorphous phase.^82^

**Table 9:** Transmission rates of water vapor for several biodegradable polymers^82^^a^

Film	Crystallinity (%)	Solubility parameter (J cm^−3^)^1/2^	Water vapor transmission rate (g per m^2^ per day)		
			*T* = 6 °C	*T* = 25 °C	*T* = 49 °C
PLA-crystalline	74	21.5	1.8	13	124
PLA-amorphous	69	21.5	3.1	21	204
PHBV, HV = 6%	62	21.4	3.5	26	245
PHBV, HV = 12%	66	22.7	27	82	333
PHBV, HV = 18%	0	22.7	54	172	1100
PCL	67	20.8	41	177	1170
Bionolle	0	—	59	330	2420
BAK 1095	0	—	134	680	3070
Cellulose acetate propionate	41	24.2	590	1700	5200
Cellulose acetate	33	25.7	1020	2920	7900

a Water solubility parameter is 47.9 (J cm^−3^)^1/2^. Abbreviations: PLA, poly(lactic acid); PHBV, poly(3-hydroxybutyrate-*co*-3-hydroxyvalerate); PCL, poly(ε-caprolactone); Bionolle, blown film containing an aliphatic polyester; BAK 1095, blown film containing poly(ester-amide).

### 2.5 PLA's degradation

**Fig. 26:**
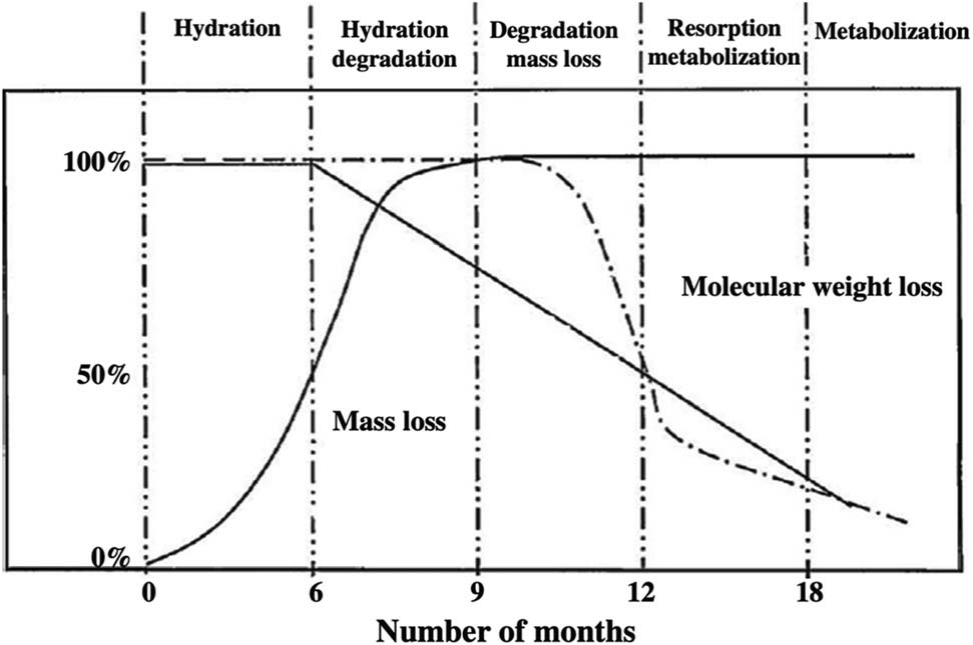
The *in vivo* degradation mechanisms for typical resorbable polymers.^95^

PLA is known for its environmental qualities and it is considered more environmentally friendly than other commonly used plastics for food packaging applications such as: PS, PE, and PP. In spite that numerous polyesters, such as PBS, PHA, and PCL are also categorized as biodegradable, yet, PLA has the advantage of producing mass production out of it because it is produced by lactic acid fermentation from sugar. Furthermore, although PBS and PCL are considered biodegradable, they are produced from petrochemical sources. At the same time, further improvement for PHA is required to enhance its production. Understanding PLA's biodegradation is of great importance for many plastic industries today in order to meet the current stringent environmental regulations. Furthermore, understanding the biodegradation behavior of PLA inside the living body is vital because PLA has been implemented in various medical applications such as sutures and implants. The erosion of PLA used in biomedical applications can be controlled by manipulating PLA's average molecular weight. PLA degradability in body fluids and tissues can be reduced by adding a d-lactide isomer. This is attributed to the fact that living body do not produce a suitable enzyme to act on d-lactic acid.^83^ The attack of polymers by external elements is the main cause behind polymer's degradation. This is due to the high stability of polymer chains which rarely undergo autocatalysis. PLA is produced from lactic acid that is obtained by the fermentation of sugars by bacteria (organic process). However, when lactic acid is converted to PLA, major changes in the chemical and biological degradation occur. PLA does not have the ability to be broken down directly and consumed by living organisms as efficiently as lactic acid itself. An important factor that affects PLA's biodegradability is its stereochemistry. Chemical bonding affects the degradation of polymers. Hydrolysis reactions are responsible for most degradation of the d-lactic acid. Exposing PLA to water for a long period is required to initiate the hydrolysis process. An approximation of the degradation times for neat polymers along with their derived copolymers are shown in Table 10. An important reason for the variation in degradation kinetics of a copolymer is that the additional monomer affects the crystallinity and reduces the steric effects.^84^ Increasing the glycolide portion has been found to increase the chain's cleavage rate. The degradation time was found to increase due to copolymerization of l-lactide with d,l-lactide. This is attributed to the oligomer d-lactic acid, that does not tend to degrade naturally by the body's enzymes. This approach can be used to extend the functionality of PLA implants in the human body. Therefore, controlling the composition of PLA's copolymers is critical for the release of drugs in the living body.^85^ Crystallization is another factor that affects polymer's degradation. The amorphous portion of PLA was found to be less resistant to degradation than the crystalline portion.^86–89^ Not only that, but it was also found that when compared to the fully amorphous regions of PLA, the amorphous regions that exist between the crystalline regions had good hydrolysis resistance.^86^ Molecular weight of a polymer has also a high impact on its biodegradability. High molecular weight polyesters are found to degrade at a slower rate.^90,91^ This is because high molecular weight molecules have greater entanglement, which means that they resist hydrolysis for chain cleavage.^83^ Other factors that influence the degradation of PLA are water uptake and acidity. Water uptake is related to hydrolytic degradation, where breakage of the polymer is achieved by the water molecules.^92^ The hydrolysis process induced by the water uptake is significant because it ensures the functionality of biopolymers in biological systems and their degradation by microorganisms. There are different factors that affect the extent of water uptake. Some of these factors are molecular weight, purity, morphology, shape of the specimen, and the processing history of the polymer. A crystal structure can decrease the capacity for water permeation. This can be accomplished by copolymerization or quenching of the polymer. Water uptake of PLA leads to the splitting of ester bonds; then, the oligomers can be assimilated by living cells. Acidity controls the rate of reaction of ester splitting through catalysis.^93^ Chu compared the degradation of poly(glycolic acid) and poly(lactide-*co*-glycolide) sutures. Results suggest that the breaking strength of an entire suture depends on the pH, particularly at high and low pH values.^94^Fig. 26 shows the *in vivo* degradation mechanisms for typical resorbable polymers such as PLA.^95^ At the start, the hydration process takes place over the first 6 months. During that time, the molecular weight remains the same, however, mass loss occurs. In order for the hydrolysis reaction with the ester bonds to be initiated, excess water is needed to penetrate the higher molecular weight structure. When enough water is accumulated in the polymer, water-soluble monomer oligomers are generated from the cleavage of the ester bonds. Lactic acid monomers are formed, which cause hydration degradation during a period of 6 to 9 months. Such monomers diffuse into the body fluids, causing substantial mass loss. Then, they are further transferred to the liver for metabolization. Throughout this stage, the lactic acid in the body's fluids goes through enzymatic degradation, however, this is only limited to the l-lactic acid because human's body does not produce the d-lactic acid enzyme. A longer period of time is required for the d-lactic acid to undergo hydrolytic degradation. Eventually, the d-lactic acid finally reduces to CO_2_ and water before being rejected from the body. As shown in Fig. 26, by the 9^th^ month, a total mass loss of the entire bioresorbable polymer took place, this was accompanied by a gradual reduction of the molecular weight.^95^

**Table 10:** Approximate degradation time of selected biodegradable polymers^96^

Polymer	Approximate degradation time (months)	Degradation products
Poly(glycolic acid)	6 to 12	Glycolic acid
Poly(l-lactic acid)	>24	l-Lactic acid
Poly(d,l-lactic acid)	12 to 16	d,l-Lactic acid
Poly(d,l-lactic-*co*-glycolic acid) (85/15)	5 to 6	d,l-Lactic acid and glycolic acid
Poly(d,l-lactic-*co*-glycolic acid) (75/25)	4 to 5	d,l-Lactic acid and glycolic acid
Poly(d,l-lactic-*co*-glycolic acid) (65/35)	3 to 4	d,l-Lactic acid and glycolic acid
Poly(d,l-lactic-*co*-glycolic acid) (50/50)	1 to 2	d,l-Lactic acid and glycolic acid

**Fig. 27:**
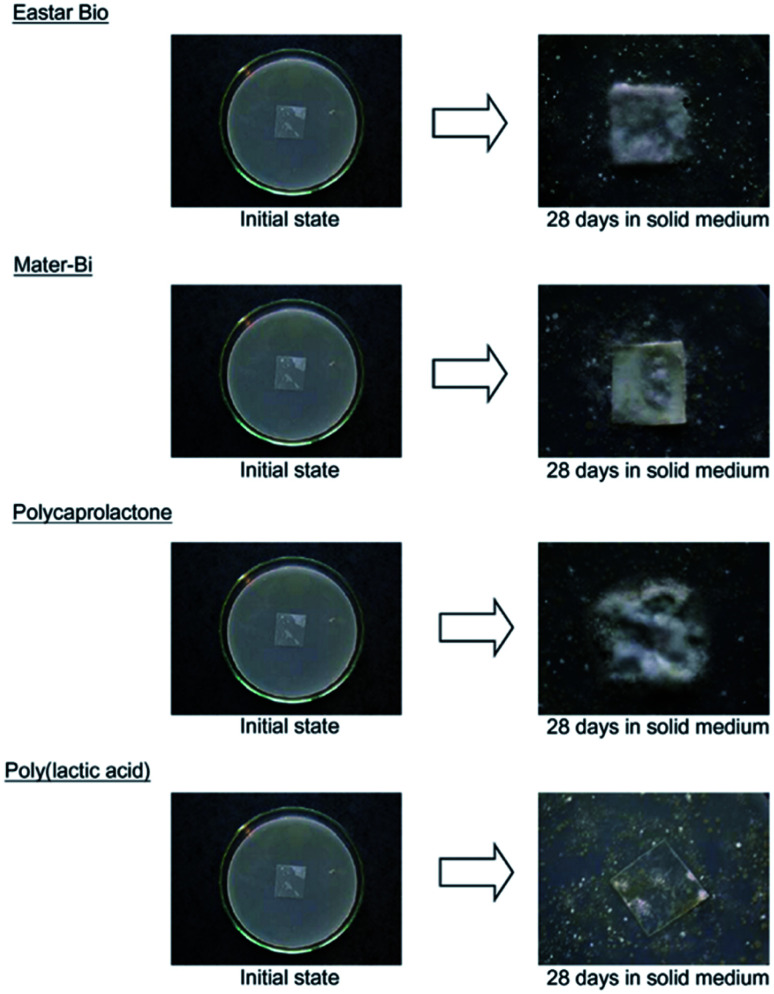
Status of different biomaterials after 28 days in solid medium.^98^

**Fig. 28:**
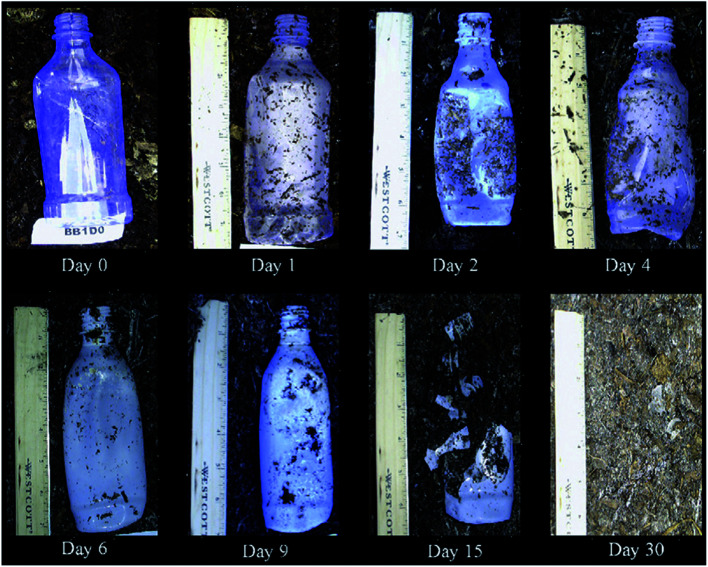
PLA bottle biodegradation's chronology in the compost pile.^101^

**Fig. 29:**
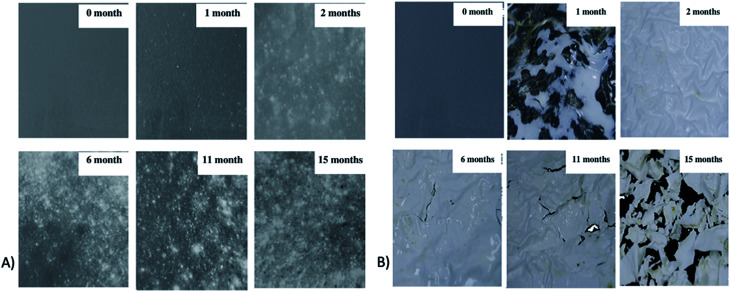
PLA sheets' degradation under: (A) wastewater treatment condition, (B) landfills conditions.^99^

In a study conducted by Torres *et al.*, various types of filamentous fungi were used to decide upon the types of microorganism strains that can affect PLA's degradation. Two analyses on dl-LA and its oligomers separately at a concentration of 10 g L^−1^ were conducted. In order to avoid biological contamination, which can produce faulty results, sterilization was undertaken. All microorganism strains were reported to actively consume lactic acid and oligomers. Out of the analyzed microorganism strains, two strains of *Fusarium moniliforme* and one strain of *Penicillium roqueforti* could totally utilize dl-LA and dl-LA oligomers as the sole carbon and energy source. On the other hand, other microorganism strains could only partially assimilate the dl-lactic acid and oligomer substances.^97^ Massardier-Nageotte *et al.* investigated the percentage of biodegradation for various polymers for different days. As per the results in Table 11, PLA had the slowest biodegradation rate among all biomaterials used in the study. Results showed that there was a lack of microorganism colonization on the surface of the PLA sample compared to the other biomaterials. Results of such observations are shown in Fig. 27. Different data suggests that PLA is durable and has the ability to resist degradation for a longer period of time compared to other biopolymers, while at the same time it has the ability to maintain its biodegradable characteristics. For some applications that require long-term use such as: woven fabrics and matting, the ability of PLA to maintain its functionality becomes of great importance.^98^ In one study, Kale *et al.* investigated the biodegradability of polylactide bottles in real and simulated composting conditions. They have used 500 ml bottles made out of PLA in their investigation. The bottles were used for packaging spring water and were manufactured by NatureWorks LLC. PLA bottles were made out of 96% l-lactide with a bluetone additive. The PLA bottles were exposed to composting burial and international standard of ASTM D5338 and ISO 14855-1 under controlled conditions. PLA bottles were completely decomposed by 30 days when they were buried in a compost pile made of cow manure, wood shavings, and waste feed. Due to the environmental heat and the microbiological action, the temperature in the compost pile reached to around 65 °C which is higher than the glass transition temperature of PLA (60.6 °C). As a result, PLA bottles were distorted in days 1 and 2. The structure of the PLA bottles remained tough until days 6–9, when a powdery texture appeared on the surface and fragmentation took place. By day 15, the PLA bottles lost their structures and large portions of the bottles had composted. By day 30, very negligible residuals from the bottle were observed. Fig. 28 shows the complete history of the degradation of the PLA bottle in the compost pile. The biodegradation of PLA sheets under various composting conditions was investigated by Rudeekit *et al.* After one moth of exposing the PLA sheets to wastewater treatment conditions, white spots on the surface were observed. It was also found that the areas impacted by the white spots had grown substantially larger over the testing period. Areas of white spots are shown in Fig. 29A. Moreover, it was found that PLA degraded more rapidly under composting plant conditions at high temperature (50–60 °C) and relative humidity higher than 60%. By day 8, the PLA sheets became brittle and started to break into small pieces. This is attributed to the fact that the degradation temperature at the land composting plant was higher than PLA's *T*_g_. Therefore, when the temperature exceeds PLA's *T*_g_, chain movement took place and that enabled the penetration of water to induce the hydrolysis reaction. PLA sheets degraded more slowly under landfill conditions than those left to degrade under composting plant conditions. This is due to the higher humidity and temperature in the composting plant conditions compared to the landfill conditions. PLA under composting plant conditions degraded completely in only 30 days. On the other hand, around 6 months was required for the PLA sheets under the landfill conditions to exhibit major fragmentation. Moreover, around 15 months was required for them to reflect some disappearance. Results of PLA sheet's degradation under the landfill conditions are demonstrated in Fig. 29B.^99^ A summary of some of PLA's composting studies along with their findings is shown in Table 12.^100^

**Table 11:** Biodegradation percentage for different biomaterials under aerobic conditions^98^^a^

Time (days)	PLA	Mater-Bi®	Eastar bio®	PCL
7	3.2	23.9	4.9	13.7
14	3.6	35.7	11.6	29.3
28	3.7	42.8	15.1	34.8

a Abbreviations: PLA, poly(lactic acid); Mater-Bi®, starch/polycaprolactone blend; Eastar bio®, poly(butadiene adipate-*co*-terephthalate); PCL, poly(ε-caprolactone).

**Table 12:** Summary of some of PLA's composting studies^100^^a^

Polymer	Degradation method	Findings	References
PLLA; in the form of non-woven fabrics and blown film; from Neste Oy	Bench-scale composting; CO_2_ measurements	99% mineralization of PLLA films and 73% and 48% mineralization of PLLA fabrics after 60 days	88
PLA; in the form of bottles; from Biota	Composting, ISO 14855, ASTM D6400 at 58 °C and 55% relative humidity	64.2% mineralization after 63 days	102
PLA; in the form of films	Composting; leaf compost rows, molecular weight measurement at 55–60 °C and 50–70% humidity	Two weeks were required for the PLA films to disintegrate physically in the compost rows; degradation rates reported were 109 173 and 68 532 *M*_w_ per week	103
PLA, laboratory synthesized PLLA	Controlled composting test (prEN14046); carbon dioxide evolution measurement	92% (±17%) biodegradation for PLLA in 202 days (56% (±5%) biodegradation in 150 days)	48
Commercial PLA; in the form of 1.5 mm extruded thickness sheets	Composting; yard waste compost; CO_2_ evolution measurement and molecular weight changes	Significant decrease in the molecular weight of PLA	104
Commercial PLA; from Mitsui Chemicals	Composting (ISO 14855-1, ISO 14855-2, enzymatic degradation at 58 °C); CO_2_ evolution measurement based on titration and gravimetric methods	91% biodegradation of PLA powder after 31 days under ISO 14855-1	105
		80% biodegradation of PLA powder after 50 days under ISO 14855-2	
Commercial PLA, in the form of bottles and delicatessen containers	Composting under real conditions (compost pile; temperature 65 °C ± 5 °C; moisture 63% ± 5%, pH 8.5 ± 0.5); visual inspection; molecular weight changes; glass transition and melting temperature; decomposition temperature	PLA delicatessen containers degraded in less than 30 days under composting conditions while PLA bottles showed very negligible residuals left from the bottle after 30 days	106
PLA	Composting at laboratory scale – simulated aerobic composting facility (as *per se* ASTM D5338)	More than 60% degradation prior to 100 days	107

a Abbreviations: PLLA, poly(l-lactic acid); PLA, poly(lactic acid).

### 2.6 PLA's recyclability, sustainability & environmental impact

Although PLA is considered as a biodegradable polymer, yet, it still has some common characteristics with petroleum-based polymers. PLA has the characteristics of thermoplastic to undergo the melt recycling method. Nonetheless, recycling PLA remains less promising; this is due to the absence of commercial volumes to cover PLA recycling plants' setup costs. Therefore, research today is directed towards establishing PLA's recycling technology in the near future. Postconsumer PLA can be either crystalline or amorphous. Semicrystalline and/or crystalline PLA can be gathered from oriented sheets, films or spun bond fibers. On the other hand, amorphous PLA can be collected from injection molded, blow molded or thermoformed parts in the form of flakes from postconsumer products. It is always a good practice to make sure that the collected PLA from postconsumer products is of high quality prior to undergoing degradation. This is because degraded postconsumer products cannot be recycled or mixed with neat PLA as they can detriment the quality of the neat polymer. Degraded PLA can be spotted *via* weaker structure, fragmentation, powdery, leakage or color fading. Crystalline as well as amorphous PLA shall be dried prior to extrusion at temperatures range of 65–85 °C and 43–55 °C, respectively. This is important so as to avoid having a reaction of PLA with water molecules which can lead to hydrolysis degradation. Moreover, another purpose of drying PLA is to avoid having a sticky low melt temperature PLA in the reclaimer of the pre-extrusion dryer. Furthermore, non-PLA materials shall never be mixed with recycled PLA resin. For example, during the recycling process of PLA bottles, it is crucial to remove the polyethylene film-printed labels attached to the bottle to avoid any undesired effects that may result from the incompatibility of the polymers components. Moreover, additives such as impact modifiers, reinforcing agents, and fillers may have an un-pleasant effect on the recycled PLA and therefore must be subjected to compatibility testing prior to the recycling process.^108^

**Fig. 30:**
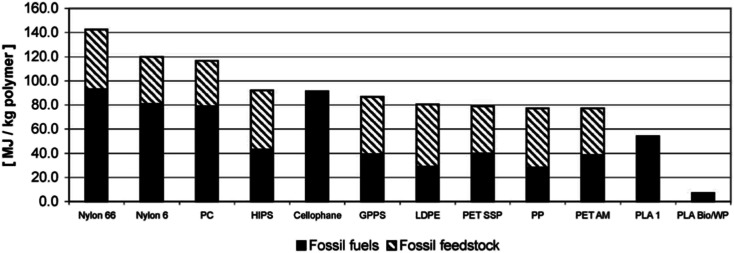
Fossil fuel energy consumption for PLA and some petroleum-based polymers. HIPS, high impact polystyrene; GPPS, general purpose polystyrene; PET SSP, polyethylene terephthalate, solid-state polymerization (bottle grade); PET AM, polyethylene terephthalate, amorphous (fibers and film grade); PLA1, PLA without adoption of biomass and wind power; PLA Bio/WP, PLA with adoption of biomass wind power.^112^

**Fig. 31:**
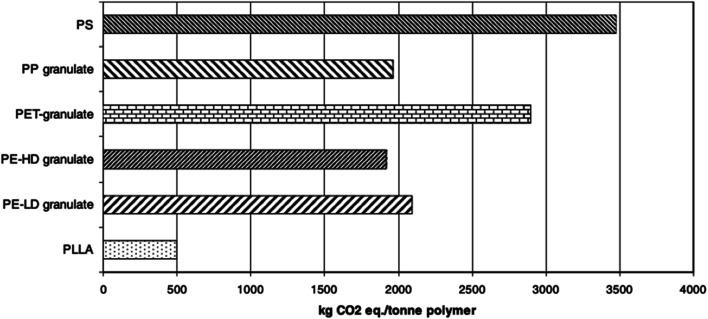
Global warming potential associated with the production of PLLA and other polymers.^115^

**Fig. 32:**
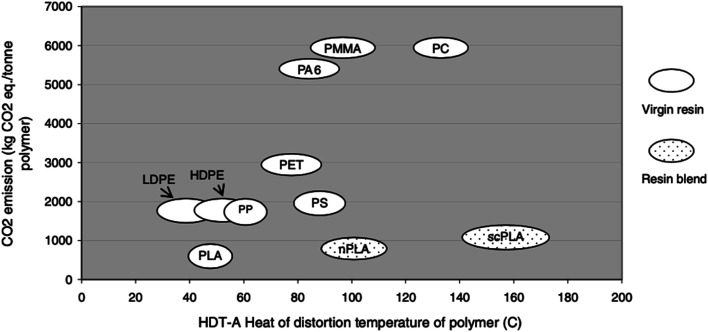
Global warming potential of polymer production as a function of the heat distortion temperature of the polymer.^115^

For the purpose of comparing the eco-profile of PLA and other materials, researchers have been relying on Life Cycle Assessment (LCA). Basically, LCA can be efficiently used to evaluate the Carbon Footprint (CF) of various materials and products. Information extracted from LCA can play an important role in minimizing the environmental impacts of products. Due to its agricultural origin, PLA is usually assumed to be an environment-friendly polymer. However, there are various elements that can influence the environmental impact of plastic products. Some of these elements may include the capability to recycle, reuse, requirement of cleaning postconsumer items, and transportation.^109,110^ Simon *et al.* investigated the LCA for aluminum cans (0.5 L and 0.33 L), PET bottles (0.5 L, 1.0 L, 1.5 L, and 2.0 L), beverage cartons (1.0 L), PLA bottle (1.5 L), and glass beverage bottles (0.33 L and 0.5 L). Results suggest that the lowest greenhouse emission was that for PLA bottle at a 66 kg carbon dioxide equivalent (CO_2_-eq.), followed by the 1.5 L PET bottle at 85 kg CO_2_-eq., and then the beverage carton at 88 kg CO_2_-eq. Nonetheless, when the materials were subjected to incineration and landfill, a tremendous increase of greenhouse emission was observed, however, greenhouse emission of PLA bottles remained the lowest with 498 CO_2_-eq. and 500 CO_2_-eq., in case of incineration and landfill respectively. Such conclusions suggest that recycling is the ultimate method to maintain a greener environment. Therefore, incineration and landfill should only be considered after products' end of life.^111^ Initially, NatureWorks LLC published the first LCA for PLA using corn as the feedstock. Using the first LCA, Vink *et al.* reported the gross energy consumption for the production of PLA. Results were unsatisfactory. Therefore, many industries saw that the production of PLA lacks justification in terms of its renewable properties and sustainability in spite of its feedstock origin being from corn. However, comparing PLA with other petroleum-based materials, PLA remains superior as it utilizes fewer fossil inputs, as illustrated in Fig. 30. Nonetheless, to improve the environment-friendly selling points of PLA, Vink *et al.* proposed substitution of fossil energy inputs with biomass/wind power.^112^ After many years of PLA production and study of renewable energy resources, Vink *et al.* reported that NatureWorks LLC was able to reach a 90% reduction in carbon emissions.^113^ That was also followed by more enhancement of the NatureWorks' PLA production process as reported by Vink *et al.*^114^ In another study conducted by Groot and Borén, an LCA for l-lactide, d-lactide, PLLA, and two PLLA/PDLA blends made from cane sugar, was carried out and then compared with that of fossil-based polymers. The global warming potential for PLLA along with other fossil-based polymers is shown in Fig. 31. It is clear that the global warming potential of PLLA is much lower than that of fossil-based polymers, and that is one of the reasons for which many companies today are switching to biomaterials. The global warming potential against the heat of distortion temperature for PLA and other polymers is shown in Fig. 32. Data for non-PLA polymers are all from the European Plastics Association and refer to virgin non-compounded materials. The data for PLA is for a virgin resin, while nPLA and scPLA are for a resin blend. The global warming potential of nPLA and scPLA are somewhat higher than that for neat PLLA, but still much more favorable than for fossil-based polymers on a weight by weight basis.^115^

**Fig. 33:**
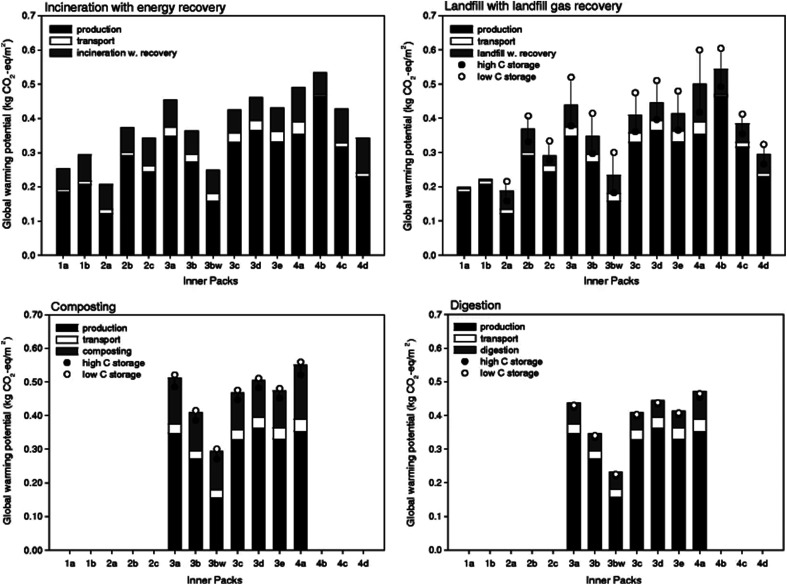
Inner packs' global warming potential for four waste treatment types.^117^

**Fig. 34:**
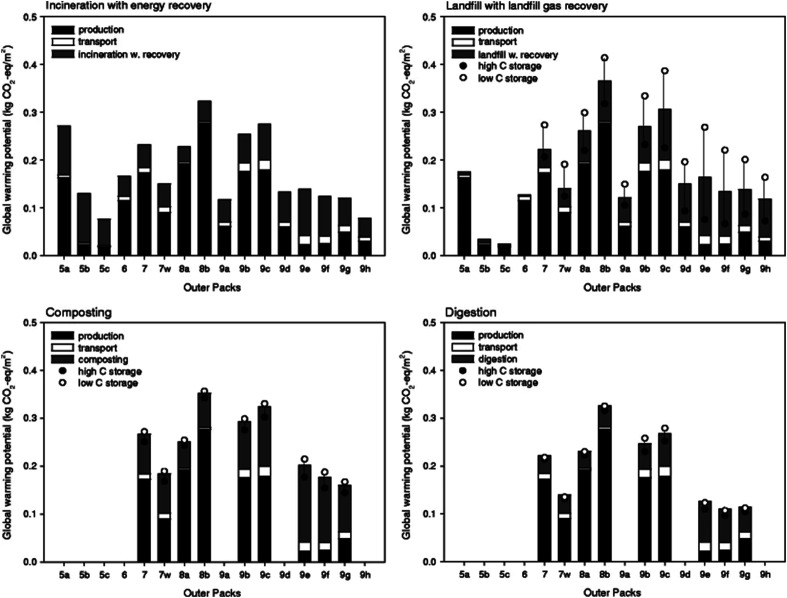
Outer packs' global warming potential for four waste treatment types.^117^

PLA is a tempting substitute for petroleum-based polymers for various applications including packaging, as well as the manufacturing of containers and cups. Petroleum-based polymers require many years to break down into harmless substances, however, PLA is fully degradable and aid in reducing the burden to the environment. In a study conducted by Vercalsteren *et al.*, four types of cups that are commonly used in small indoor as well as large outdoor public events in Belgium were analyzed: the reusable polycarbonate cup (PC), the one-way polypropylene cup (PP), the one-way PE-coated cardboard cup, and the one-way polylactide cup (PLA). The functional unit was defined as the recipients needed to serve 100 L of beer or soft drink at a small-scale indoor event (2000–5000 visitors) and a large-scale outdoor event (more than 30 000 visitors). Factors including production of the cups, consumption stage (at the events), and the processing of cups wastes were taken into consideration. By comparing the environmental impacts of the four types of cups on both of the small indoor and large outdoor events, it was concluded that none of the cup systems has the highest or the lowest environmental score for all environmental impact categories considered in the study (carcinogens, ecotoxicity, fossil fuels, *etc.*). Therefore, it was not possible to draw a straightforward conclusion about the selection of the most favorable cup system (neither at small nor large events) with regard to the environment. Furthermore, the various events' size had also an impact on the eco-efficiency of the cups. For example, the lowest environmental impact in small public events was reported when the PC cup was utilized. This can be attributed to the reusable PC. Therefore, the PC cups were washed by hand which lowered the use of water and detergent throughout the cleaning process. However, at large public events, the environment impact for the PC cups was higher. This was due to the fact that there was more frequent cleaning for the PC cups which made them wear out faster, hence, they were regularly replaced. The highest eco-indicator points were for PLA cup, yet, for long-term applications, PLA still remains competitive due to its current technology development still being at the beginning stage. Furthermore, some of the environmental aspects associated with PLA such as acidification/eutrophication and dependence on fossil fuels could be further reduced under the appropriate measures. The successful implementation of such measures resulted in an almost 20% decrease in the eco-indicator points for the NatureWorks' second-generation Ingeo (PLA6) when compared to their first-generation type PLA cup (PLA5).^116^ In an investigation conducted by Hermann *et al.*, the authors compared the use of different biomaterials (paper, polylactic acid, bio-based polyethylene, and a bio-based polyester) as well as conventional polymers (PP and PE) in the fabrication of 1 m^2^ that is mostly laminated and printed packaging film. The impact assessment for non-renewable energy use, total energy use, global warming potential, depletion of abiotic resources, photo-oxidant formation, acidification, eutrophication, water use, and land use were presented. The materials for the films and laminates were selected based on their collaboration with a multinational food producer and its film suppliers and converters. The two criteria for selection were that films and laminates must consist of at least in part of bio-based materials and that they must exhibit or are expected to have comparable barrier properties as the current materials used in the market. Different inner packs (1a–4d in Table 13) as well as outer packs (5a–9h in Table 13) alternatives were analyzed. Inner packs materials are chosen to have a good water and oxygen barriers because they are in a direct contact with the food. Outer packs are not in direct contact with the food, and they only serve as containers or bags for the inner packs. Fig. 33 shows the different global warming potential for the various inner packs. The global warming potential was calculated by adding all emissions of fossil greenhouse gas emissions and subtracting the biogenic carbon that is physically embedded in the product. Both fossil and biogenic emissions of greenhouse gases from the waste treatment stages were considered. Among all the biodegradable laminates for inner packs, the one double-layer PLA film (no. 3bw–3bw refers to PLA that was produced using wind energy instead of fossil energy) scored best for composting and digestion. Overall, the environmentally most attractive outer packs are bio-based PE (no. 5c), paper/PP laminate (no. 9a), paper/EVA (no. 9h), paper/bio-based polyester (no. 9g) and to a somewhat lesser extent also paper/petrochemical PE (no. 9d) (see Fig. 34). According to the study, inner and outer packs that contain PLA film produced without using wind energy, offer no significant environmental advantages. Nonetheless, when PLA's future technology is taken into consideration or if wind credits are assigned, PLA laminates become environmentally comparable with the reference material in the investigation.^117^

**Table 13:** Laminates included in the study; biodegradability is indicated by an asterisk. Reference materials are 1a and 1b for inner packs and 5a and 6 for outer packs. For inner packs: PP laminates are 1, PP hybrids are 2, PLA laminates are 3, paper laminates are 4. For outer packs: PE films are 5, PP film is 6, PLA film is 7, cellulose films are 8 and paper laminates are 9 (ref. 117)^b^

Material type	Number	Material type	Number
OPP/PE/MOPP	1a	PE	5a
OPP/PE/MOPP	1b	Bio-based PE	5b
Paper/PE/MOPP	2a	Bio-based PE	5c
Cellulose/PE/MOPP	2b	OPP	6
PLA/PE/MOPP	2c	PLA	7*
MPLA/PLA/PLA	3a	Cellulose	8a*
PLA/AlO_*x*_ coated PLA	3b*	Cellulose	8b*
PLA/SiO_*x*_ coated PLA	3c*	Paper/OPP	9a
PLA SiO_*x*_ coated/SiO_*x*_ coated PLA	3d*	Paper/PLA	9b*
MPLA/MPLA	3e*	Paper/PLA	9c*
Paper/SiO_*x*_ coated PLA/PLA	4a*	Paper/PE	9d
Paper/aluminum/PLA	4b	Paper/BBP	9e*
Paper/MPET/peelable^a^ PP	4c	Paper/BBP	9f*
Paper/MPET/peelable PE	4d	Paper/BBP	9g*
		Paper/EVA	9h

a The layer can be easily removed, manually, from the laminate.

b Abbreviations PP, poly(propylene); OPP, oriented poly(propylene); MOPP, metallised oriented poly(propylene); PE, poly(ethylene); PLA, poly(lactic acid); AlO_*x*_, aluminum oxide; MPLA, metallised PLA; SiO_x_, silicone oxide; MPET, metallised poly(ethylene terephthalate); BBP, bio-based polyester, that is not further specified as per the producer's request; EVA, ethyl vinyl acetate.

**Fig. 35:**
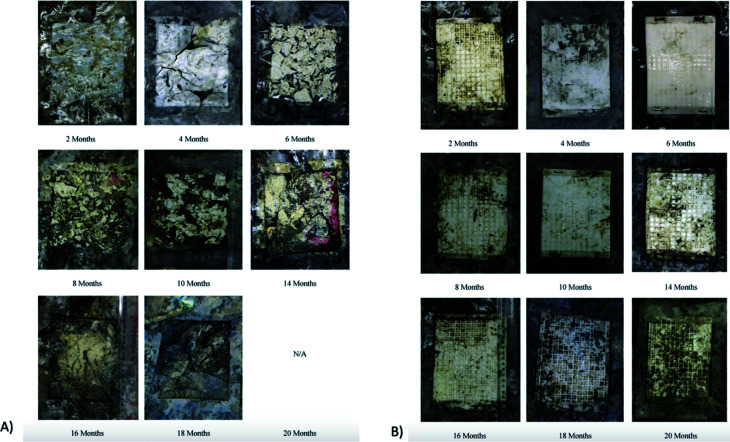
The chronology under landfill conditions for up to 20 months for: (A) PLA sheets, (B) PS sheets.^118^

In their investigation, Leejarkpai *et al.* discussed the importance of considering the role of land-use change on the environmental impact of PLA. Land-use change is an element that need to be considered when lands are converted to crops. This can involve significant amount of processing including decomposition, nitrification/denitrification, and combustion. All such processes contribute in way or another to the global warming. The study showed that the highest CO_2_ emissions are related to that of PLA with land-use change consideration followed by PET, while the lowest emissions were reported for PS. However, under landfill conditions, the study reported that PLA showed a superior biodegradation property compared to PS. The structure of PLA sheets was subjected to biodegrading and it was broken down after 6 months burial in landfill conditions as shown in Fig. 35A. On the other hand, no significant difference in the structure of PS sheets was spotted even after 20 months as illustrated in Fig. 35B. Hence, the superior PLA's biodegradation characteristics plays an important role in reducing the plastic pollution due to nondegradable plastics.^118^

## 3. Polyhydroxyalkanoates (PHAs)

**Fig. 36:**
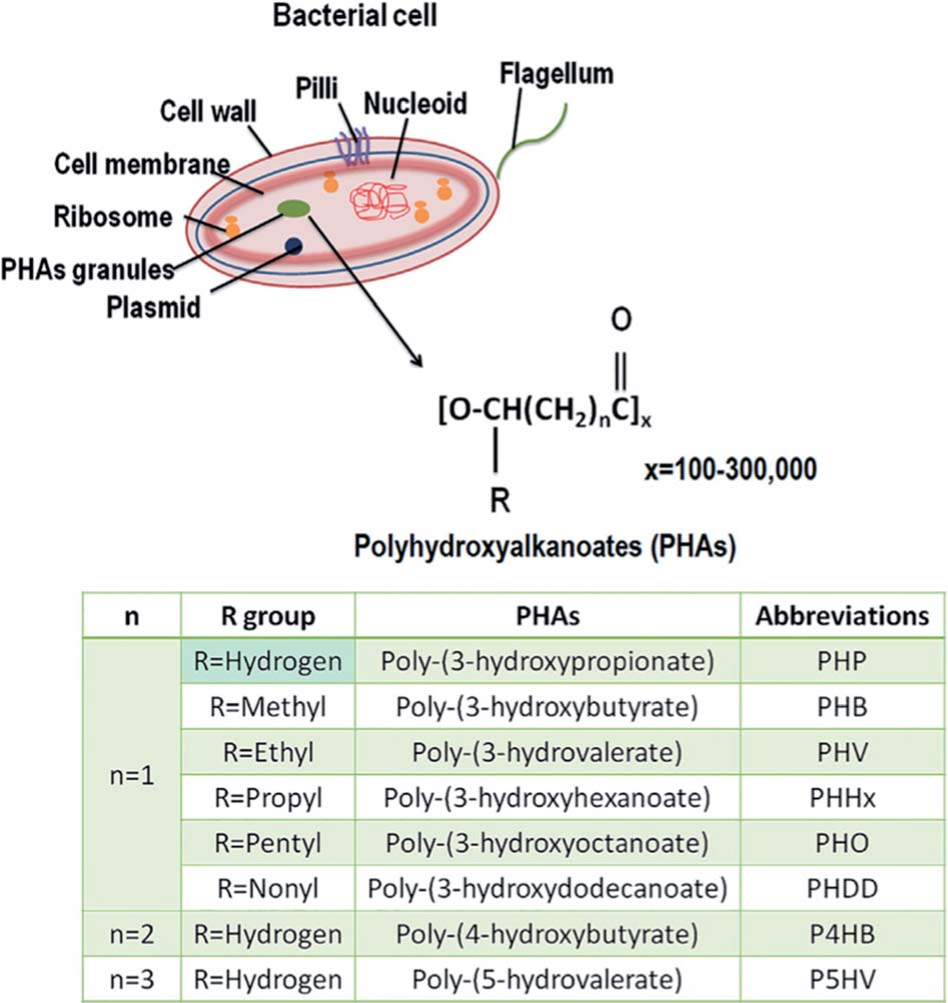
PHAs' chemical structures along with their derivatives.^137^

**Fig. 37:**
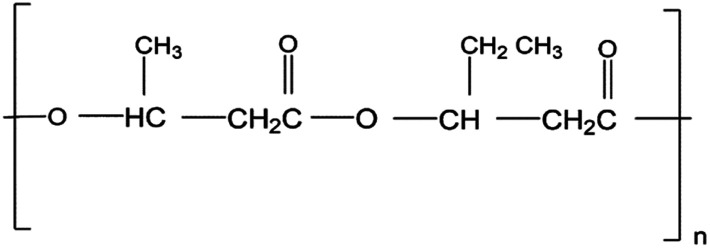
The chemical structure of the poly(3-hydroxybutyrate-*co*-3-hydroxyvalerate) copolymer.^136^

PHAs can be defined as a family of intracellular biopolymers that are synthesized *via* various bacteria as intracellular carbon and energy storage granules. This family of biopolymers is produced by fermentation from natural resources, specifically, sugar or lipids. PHAs composed of hydroxyalkanoate (HA) units, arranged in a basic structure that is obtained through bacterial fermentation. PHAs are considered as opening doors for a sustainable future.^119^ PHAs general characteristics include, but not limited to, water insolubility, relative resistant to hydrolytic degradation, biocompatibility and suitability for medical applications, as well as nontoxicity. Although, PHAs are not water soluble, they are still degradable and biocompatible. In addition, PHAs are considered less sticky than other polymers once heated, and they sink in water which facilitates their anaerobic biodegradation in sediments. Among the most commercialized and produced biopolymers, PHAs stand out as a tempting sustainable alternative. This is attributed to their ability to be transformed into water and carbon dioxide if oxygen is present. They can also be transformed into methane under anaerobic conditions, *via* microorganisms present in water and soil.^120^ Due to their biodegradable nature, PHAs are intended to replace synthetic non-degradable polymers for various applications, such as: packaging, fast food, medicine, biomedical, and agricultural applications. Moreover, the fact that they can be produced from renewable resources made them an excellent choice for short term packaging. Furthermore, they are considered to be biocompatible in contact with living tissues and they can be safely used for biomedical applications such as: tissue engineering and drug encapsulation.^121–123^ In addition, due to their biodegradability, renewability, and potentially useful water vapor barrier properties, PHAs-based films have attracted many food packaging industries. PHAs can be processed well by injection molding. The importance of PHAs is also attributed to their null toxicity, and high biocompatibility with many various types of cells.^124–126^ Nonetheless, the energy intensive extraction and purification step required for processing PHAs make them one of the most expensive bio-based plastics.^127^ A variety of PHAs' monomers as well as co polymers can be obtained based on the metabolism of the microorganism and the carbon substrates.^121^ PHAs can be degraded *via* abiotic degradation, that does not need the presence of enzymes to catalyze the hydrolysis. During the biodegradation process, the residual products are degraded by the enzymes until final mineralization. Generally, PHAs are classified by the different number of carbons in their repeating units into short chain length (sCL-PHA) and medium-chain-length (mCL-PHA), sCL-PHA have 4 or 5 carbons in their repeating units, besides 6 or more carbons in the repeating units for mCL-PHA. Examples of sCL-PHA are: P(3HB) and P(4HB), while P(3HHx), P(3HO), and P(3HHx-*co*-3HO) are examples of mCL-PHA.^128^Fig. 36 shows the chemical structures of PHAs and their derivatives, R can be hydrogen or hydrocarbon chains of up to around C16 in length. Table 14 shows the main PHAs homopolymer. PHAs are mostly made from saturated and unsaturated hydroxy-alkanoic acids. Based on the type of monomer, PHAs can be homo-, co- and terpolymers. Various PHAs with different properties can be obtained from variety of monomers and by varying constitutional isomerism. Table 15 shows various poly(hydroxybutyrate-*co*-hydroxyalkanoates) copolyesters. These copolymers usually have custom sequence, and they differ in the type and the proportion of monomers. For instance, poly(3-hydroxybutyrate-*co*-3-hydroxyvalerate) or P(3HB-*co*-3HV) is based on a custom arrangement of two monomers with R as methyl and with R as ethyl. Poly(3-hydroxybutyrate-*co*-3-hydroxyhexanoate) consists of two monomers with R = methyl and propyl.^24,30^ The most common and simple representative of PHA is poly(3-hydroxybutyrate) or PHB.^129^ PHB is highly crystalline with good gas barrier properties.^130,131^ One of PHB's drawbacks is related to its very low resistance to thermal degradation. The melting temperature of PHB is between 170–180 °C which is close to its degradation temperature that is around 270 °C.^131,132^ PHB has been reportedly suffered from poor mechanical properties, mainly on account of its high fragility. Therefore, its use in many applications is hindered today.^133–135^ On the other hand, out of all PHAs' copolymers, one of the most promising materials is the poly(3-hydroxybutyrate-*co*-3-hydroxyvalerate) biopolymer abbreviated usually as PHBV or PHBHV, originated from the insertion of 3-hydroxyvalerate (HV) units to the PHB biopolymer. PHBV is an aliphatic polyester with the chemical structure as illustrated in Fig. 37.^136^ International companies with interests in the research and production of PHAs both in the past and present are summarized in Table 16.^30^

**Table 14:** Major PHAs homopolymers^30^

Chemical name	Abbreviation	Values of *x*	R group
Poly(3-hydroxypropionate)	P(3HP)	1	Hydrogen
Poly(3-hydroxybutyrate)	P(3HB)	1	Methyl
Poly(3-hydroxyvalerate)	P(3HV)	1	Ethyl
Poly(3-hydroxyhexanoate) or poly(3-hydroxycaproate)	P(3HHx) or P(3HC)	1	Propyl
Poly(3-hydroxyhexanoate)	P(3HH)	1	Butyl
Poly(3-hydroxyoctanoate)	P(3HO)	1	Pentyl
Poly(3-hydroxynonanoate)	P(3HN)	1	Hexyl
Poly(3-hydroxydecanoate)	P(3HD)	1	Heptyl
Poly(3-hydroxyundecanoate)	P(3HUD or P(3HUd)	1	Octyl
Poly(3-hydroxydodecanoate)	P(3HDD) or P(3HDd)	1	Nonyl
Poly(3-hydroxyoctadecanoate)	P(3HOD) or P(3HOd)	1	Pentadecanoyl
Poly(4-hydroxybutyrate)	P(4HB)	2	Hydrogen
Poly(5-hydroxybutyrate)	P(5HB)	2	Methyl
Poly(5-hydroxyvalerate)	P(5HV)	3	Hydrogen

**Table 15:** PHAs abbreviations for their homopolymers and copolymers^30^

Full abbreviation	Short abbreviation	Structure
P(3HB)	PHB	Homopolymer
P(3HV)	PHV	Homopolymer
P(3HB-*co*-3HV)	PHBV	Copolymer
P(3HB-*co*-3HHx)	PHBHx	Copolymer
P(3HB-*co*-3HO)	PHBO	Copolymer
P(3HB-*co*-3HD)	PHBD	Copolymer
P(3HB-*co*-3HOd)	PHBOd	Copolymer

**Table 16:** A summary of international companies (past and present) involved in PHAs' research and production^59^

Types of PHA	Company	Production scale (tons per year)	Period	Applications
Several PHAs	Metabolix, USA	Unk.	1980–2016	Packaging
	Tepha, USA	PHA medical implants	1990 to present	Medical bioimplants
	ADM, USA (with Metabolix)	50 000	2005–2016	Raw materials
	P&G, USA	Unk.	1980–2005	Packaging
	Danimer scientific	Unk.	2007 to present	Varies
	CJ CheilJedang Corporation	Unk.	2016 to present	Varies
	Meredian, USA	10 000	2007 to present	Raw materials
	Kaneka, Japan (with P&G)	Unk.	1990 to present	Packaging
	Shantou Lianyi Biotech, China	Pilot scale	1990–2005	Packaging and medicals
	Shenzhen O'Bioer, China	Unk.	2004 to present	Unk.
	Shandong Lukang, China	Pilot scale	2005 to present	Raw materials and medicals
PHA (unclear)	Bio-On, Italy	10 000	2008 to present	Raw materials
	Yikeman, Shandong, China	3000	2008 to present	Raw materials
PHB	Chemie Linz, Austria	20–100	1980s	Packaging and drug delivery
	BTF, Austria	20–100	1990s	Packaging and drug delivery
	Biomers, Germany	Unk.^a^	1990s to present	Packaging and drug delivery
	Mitsubishi, Japan	10	1990s	Packaging
	Biocycles, Brazil	100	1990 to present	Raw materials
	Tianjin Northern food, China	Pilot scale	1990s	Raw materials
	Jiangsu Nan Tian, China	Pilot scale	1990 to present	Raw materials
PHB, PHBV	BASF, Germany	Pilot scale	1980–2005	Blending with Ecoflex
	Monsanto, USA	Plant PHA production	1990s	Raw materials
PHBV	ICI, UK	300	1980–1990	Packaging
	Zhejiang Tian An, China	2000	1990 to present	Raw materials
PHBHHx	Jiangmen Biotech Center, China	Unk.	1990s	Raw materials
P3HB4HB	Tianjin green Bioscience (with DSM)	10 000	2004 to present	Raw materials and packaging

a Unknown. Abbreviations: PHA, polyhydroxyalcanoates; PHB, polyhydroxybutyrate; PHBV, poly(3-hydroxybutyrate-*co*-3-hydroxyvalerate); PHBHHx, poly(3-hydroxybutyrate-*co*-3-hydroxyhexanoate); P3HB4HB, poly[(*R*)-3-hydroxybutyrate-*co*-4-hydroxybutyrate].

### 3.1 PHAs' synthesis

**Fig. 38:**
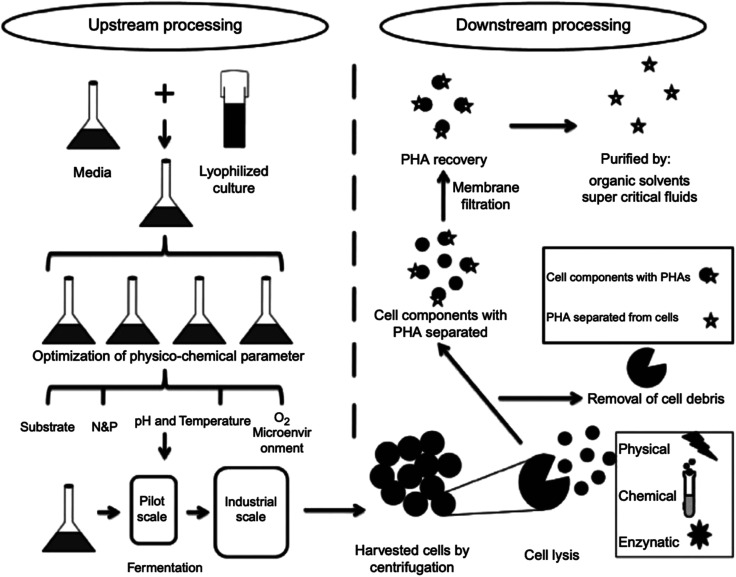
PHA synthesis.^128^

**Fig. 39:**
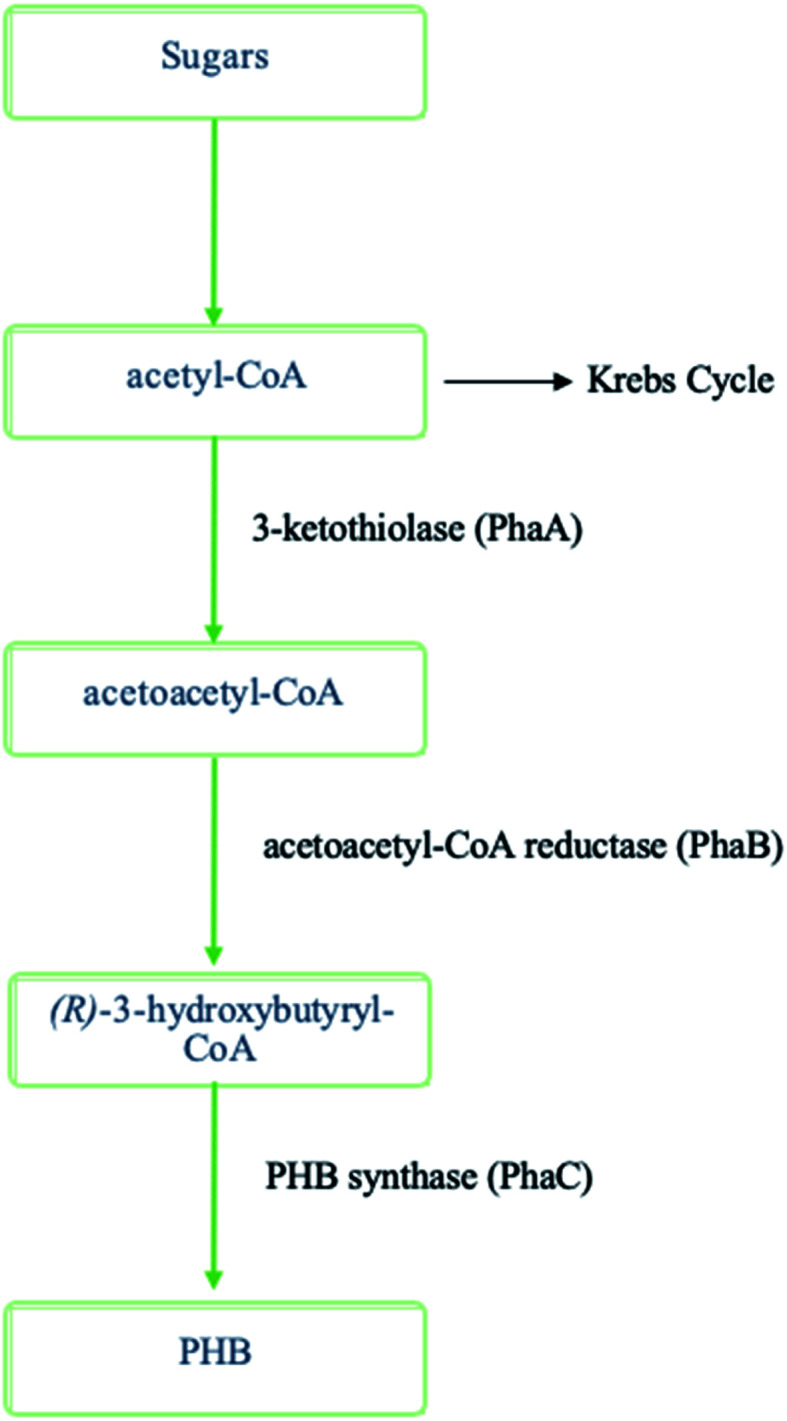
Metabolic route to polyhydroxybutyrate (PHB).^147^

PHAs can be produced from plants and bacteria. However, high level of PHAs production is currently not feasible *via* plant cells, this is attributed to the negative impact that high levels of polymers have on plants growth. Research is focused today on overcoming such concerns.^138^ On the other hand, high level of PHAs (up to 90% w/w of the dry cell mass) can be obtained *via* bacteria.^139^ Various prokaryotic organisms store PHA from 30% to 80% of their cellular dry weight.^140,141^ PHAs are packed in the cytoplasm of a wide variety of both Gram-positive and Gram-negative microorganisms as granular inclusions. This takes place when these microorganism experience conditions of nutritional deficiency from elements, such as magnesium, nitrogen, sulphur, and phosphorus in the presence of excess carbon.^34^ While the lack of nitrogen is the most common limitation, for some bacteria, the lack of oxygen is the most effective one.^142^ The stress conditions encountered by bacterial cells leads to an accumulation of PHA to store energy and carbon.^139,143^ These stress conditions are usually generated *in vitro* by subjecting the bacteria to nutrient limitations. There are more than 300 various microorganisms that can produce PHAs as natural energy reserves. Factors such as: microorganism's production rates, the stability and biological safety of the microorganism, PHA extractability, and the molecular weight of the agglomerated PHA will all decide upon which type of microorganism is the most suitable for a certain application. The process of PHA synthesis from bacteria is illustrated in Fig. 38. Inoculation is the initial step in the bacterial fermentation process. During this step, the bacteria needed for the subsequent metabolization process increases in numbers and grow in an aqueous environment supplemented with air under optimum physical conditions and a balanced nutrition supply. The following step consists of actual PHA synthesis under conditions that do not favor the growth and multiplication of bacteria, then the PHAs are stored in intracellular inclusion bodies. PHAs' molecular weights can range from 100 000 to 500 000 g mol^−1^. Nonetheless, higher molecular weight that are more than 1 000 000 g mol^−1^ are obtained under special conditions. Typically, the complete bacterial fermentation process takes around 2 days.^34,144^ A PHA granule's surface is coated with a layer of proteins and phospholipids. The size and number of PHA granules are affected by a class of proteins called phasins.^145,146^ PHB is the most studied out of the PHAs family. Acetyl-coenzyme-A (acetyl-CoA) is produced by bacteria in their metabolism. Conversion of acetyl-CoA into PHB is accomplished through three biosynthetic enzymes as shown in Fig. 39.^147^ The first step involves the formation of acetoacetyl-CoA by combining 3-ketothiolase (PhaA) with two molecules of acetyl-CoA. Next, the reduction of acetoacetyl-CoA by NADH to 3-hydroxybutyryl-CoA is done using acetoacetyl-CoA reductase (PhaB). Lastly, PHB synthase (PhaC) polymerizes 3 hydroxybutyryl-CoA to PHB while coenzyme-A is released. For the polymerizing enzyme, only (*R*)-isomers are accepted as substrates.^148^ Throughout regular bacterial growth, an inhabitation of the 3-ketothiolase is done by free coenzyme-A coming out of the Krebs cycle. However, the lack of non-carbon nutrient leads to restricting the entry of acetyl-CoA into the Krebs cycle. Therefore, excess acetyl-CoA is directed into PHB biosynthesis.^147,149^

### 3.2 PHAs' physical & thermal properties

Compared to other biodegradable polyesters, PHB has a high melting point, between 173 °C and 180 °C, while its glass transition temperature is roughly 5 °C. PHB has a narrow melt processing window higher than its melting temperature. A significant polymer degradation and therefore molecular weight drop can be noticed when processing PHB above its melting point.^150–152^ Moreover, PHB is highly crystalline and suffers from high brittleness and stiffness compared to conventional thermoplastics. PHB's properties can be compared with those of synthetic thermoplastics such as isotactic PP.^153,154^ Despite some of the enhancements it offers over PHB as shown in Table 17, PHBV exhibits high fragility, considerable hydrophobicity, low impact resistance, and poor thermal stability compared to petroleum-based polymers.^155^ In addition, PHBV is a rigid and rather brittle biopolymer, has a melting temperature lower than PHB, and can be dissolved in chlorinated solvents.^156^ In order to overcome the shortcomings of PHB, the incorporation of other monomers such as 3-hydroxyhexanoate (HHx) or 3-hydroxyvalerate (3-HV) during the polymerization of PHB offers a good solution.^157–159^ However, PHBV provides some improvements because it is more flexible than PHB and has a lower melting temperature. This allow PHBV to have a wider window of processing temperatures.^122,158,160–164^ At the time that PHAs homopolymers are not considered ideal for processing conditions, and PHB is somewhat difficult to process, processing PHBV is easier. Compared to PHB homopolymer, the PHBV copolymer has better mechanical properties such as toughness, impact resistance, flexibility and manufacturability. The performance of the PHBV as well as its material properties can be significantly altered by varying the valerate content (3HV) monomer. Therefore, the physical and mechanical properties of PHBV greatly depend on the 3HV content in the copolymer.^165^ As the 3HV fraction increases, the degradation rates of PHBV increase because the crystallinity of PHBV decreases with increasing 3HV content.^166^ With increasing the 3HV composition also, PHBV's melting point decreases.^167^ Thus, it is vital to select the PHBV with the desired 3HV content depending on the intended application. An increase of the HV content from 0% to 50% can result in lowering the melting and the glass transition temperatures of the resulted polymer in a significant way. Furthermore, increasing the HV content can induce an increase in impact strength, however, tensile strength, crystallinity, degradation, and water permeability decrease.^154,168–170^ High valerate content PHBV offers significant enhancement in the physical properties, specifically, in terms of increased flexibility, low crystallinity, and decreased crystallization rate.^120,163^ However, PHBV losses its Young's modulus, yield strength, and became rubbery in nature as a result of increasing the proportion of HV content. Similar to PLA, PHAs are sensitive to processing conditions. A rapid decrease in viscosity and molecular weight is obtained under extrusion. This can be attributed to macromolecular chain cleavage resulted from increasing the temperature, the shear level, and/or the residence time.^170^ Some of the average properties of PHAs are shown in Table 18.^24^ PHAs' crystallinity varies from highly crystalline to flexible (0–60%).^171,172^ The isostatic PHB is a brittle homopolymer and a highly crystalline.^173,174^ PHB's brittleness can be attributed to the formation of spherulites and secondary crystallization during its storage at room temperature.^175,176^ The secondary crystallization of PHB results in the reorganization of crystal lamellae. This strongly restricts the amorphous chains of polymer between the crystals.

**Table 17:** Physical properties of PHB and PHBV^177^^a^

Properties	PHB	PHBV
Density (g cm^−3^)	1.25	1.25
Young's modulus (GPa)	0.93	2.38
Elongation at break (%)	5.2–8.4	1.4
Traction resistance (MPa)	21	25.9
Glass transition temperature (°C)	(−)10	(−)1
Fusion temperature (°C)	161	153

a Abbreviations: PHB, polyhydroxybutyrate; PHBV, poly(3-hydroxybutyrate-*co*-3-hydroxyvalerate).

**Table 18:** Average PHAs' properties^24^

Properties	Value
Tensile strength (MPa)	15–40
Young's modulus (GPa)	1–2
Elongation at break (%)	1–15
Glass transition temperature (°C)	2
Melting temperature (°C)	160–175
Degree of crystallinity (%)	40–60
Water vapor transmission rate (g mm per m^2^ per day)	2.36
Oxygen transmission rate (cc mm per m^2^ per day)	55.12

The crystallinity of PHBV was found to decrease slightly when hydroxyvalerate content increases. This might be attributed to the greater difficulty to accommodate polymer chains in the crystalline phase as a result of the presence of the ethyl group in the monomer hydroxyvalerate.^178^ For the purpose of accelerating the uniform crystal formation as well as to improve a polymer's toughness and softness, a nucleating agent and plasticizer are added. They work to reduce the polymer's crystallinity, diminish the intramacromolecular bonding and assist conformational changes during polymer melting.^179,180^ Some of the common plasticizers that are incorporated with PHAs to reduce its crystallinity include laprol 5003, laprol 503,^181^ dioctyl sebacate (DOS),^162,181^ dibutyl sebacate (DBS),^180,181^ soybean oil, epoxidized soybean oil, dibutyl phthalate (DBP), triethyl citrate (TEC),^180^ polyethylene glycol (PEG).^181–184^ Moreover, acetyl-tri-*n*-butyl citrate (ATBC), tributyl citrate (TBC), salicylic ester, terpene d-limonene (LIM) have been also used.^185^ One of the limiting factors in the processing and application of PHAs is their thermal instability.^164,186^ Different attempts have been investigated to try to enhance the thermal stability of both, PHB and PHBV.^187–190^ PHAs' thermal degradation near the melting point takes place due to the non-radical random chain-scission reaction and it is believed that the depolymerization of the macromolecular chains is the controlling step.^151,190,191^ The thermal degradation of PHAs becomes particularly important at temperatures exceeding 200 °C.^187^ PHA's glass transition temperature is between −52 °C to 4 °C while its melting temperature is non observable to 177 °C. PHA's thermo-degradation temperature is in the range of 227–256 °C.^172,187,192^Table 19 shows thermal properties of PHB and PHBV along with polyolefins polymers. Many studies in literature have agreed that a reduction in the *T*_m_ is observed upon increasing the HV content in a PHBV copolymer. Therefore, this results in increasing the processing temperature window and maintaining degradation rates within acceptable limits.^154,188,193–196^ In their studies,^178,197^ the authors reported a drop in the *T*_m_ for PHB from 176 °C to 158 °C with 22 wt% HV content. In another study, the same drop in the melting temperature was obtained with only 8 wt% HV content.^196^ In a different investigation, PHBV with 30 wt% HV exhibited a melting temperature decrease of 70 °C.^188^ On the other hand, a stronger influence on PHAs' thermal degradation can come from the initial molecular weight.^196^ Degradation effect because of random chain-scission is observed to be higher for PHAs with lower molecular weight. Different studies in literature investigated the effect of blending PHAs with other biopolymers to enhance PHAs' thermal stability.^157,175,198–201^ Enhancing PHAs' thermal stability is also possible through the incorporation of inorganic nanofillers including montmorillonites (MMTs) and Layered Double Hydroxides (LDHs).^153,173,186,195,196,202–206^ It is believed that such enhancement is attributed to the dispersed silicate layers acting as a barrier to oxygen as well as to the volatiles produced during thermal decomposition.^153,202,203,207^ In one study, Choi *et al.*, found that the decomposition onset temperature increased from 252 to 263 °C due to the addition of 1–3 wt% Cloisite® 30B to PHBV.^153^ In another study, the incorporation of 5 wt% Cloisite® 15A nano clay to PHBV was found to increase the temperature corresponding to 50% degradation of neat PHBV.^203^ Nanocomposites' thermal stability is highly influenced by the degree of dispersion. Agglomerates can cause local accumulation of heat and trigger more rapid thermal decomposition.^173,202^ Various factors such as, processing conditions, the amount and the nature of clay can influence the dispersion of nano clays within a polymer matrix. Furthermore, the nanofiller content can also be vital because above a certain loading, agglomerates can occur. Lim *et al.* reported a higher decomposition onset temperature for PHB with 3 wt% organo-modified montmorillonite (OMMT) than that for unreinforced PHB. Yet, a drop in the nanocomposites' thermal stability was reported by further nanofiller addition.^202^ Erceg *et al.* found that 5 wt% OMMT is the load limit for increasing PHB's thermal stability.^186^ On the other hand, the incorporation of up to 10 wt% OMMT to PHBV, was found to enhance the thermal stability despite the fact that agglomerates at the highest loading was observed.^208^ It is believed that the presence of aluminum Lewis acid sites in the silicate layers improves PHB's thermal degradation *via* catalyzing the hydrolysis of ester linkages. This phenomenon is more prominent at higher loading levels.^186^ PHAs' decomposition temperature was increased due to the incorporation of 1.2–3.6 wt% OMMT. Nonetheless, when 2.2 wt% hydrophilic unmodified MMT was added to PHAs, a drop in the decomposition temperature was reported. This decrease in the decomposition temperature might be attributed to poor dispersion.^209^ Clay organomodifiers such as quaternary ammonium salts can have as strong catalyst impact on PHB's and PHBV's thermal degradation.^210–212^ PHB's thermal stability as well as its degradation kinetics were studied.^173,186^ Two types of nano clays, Cloisite® 30B and Cloisite® 25A were used in the investigation. Results showed that two regions of pure PHB and PHB/Cloisite® 30B nanocomposites were subjected to the isothermal degradation. These two regions were categorized as low and major mass loss, respectively. Melt processing of polymers is one of the most commonly used technique for the processing of polymer nanocomposites. To attain nano clay exfoliation during extrusion, high shear rates are normally required, however, they can contribute to the degradation of PHAs.^195^ Some studies on the incorporation of LDH with PHA were reported. In one of the studies, the thermal degradation mechanism of PHB containing 2% and 5% poly(ethylene glycol) phosphonate (PEOPA)-modified LDH (PMLDH) was investigated by Wu *et al.* Results of this study showed that there was no improvement in the thermal stability of the nanocomposites due to the addition of the organically modified LDH. However, samples containing 5% PMLDH exhibited a decomposition temperature of 240.2 °C compared to 263.6 °C for neat PHB. Therefore, the thermal degradation of PHB might have been catalyzed by the organic modifier.^204^ Hence, although the incorporation of nanofillers might seem like an attractive option to enhance the thermal stability of PHAs, yet, factors like the filler type and content, the organomodifier, as well as the processing conditions have to be carefully selected. In addition, thermal stability of PHAs' end products at lower temperatures during storing or transportation of PHA-based packaging would also be significant. For example, stacked packaging trays made out of PLA can lose mechanical stability and collapse at temperatures above the glass transition temperature, which is typically in the range of 50–59 °C.^213,214^ In contrast, processed PHB can be highly crystalline and shows no such softening at temperatures likely to be encountered during storage and transport.^215^

**Table 19:** Thermal properties of PHAs and some polyolefins^194^^a^

Polymer	Glass transition temperature (°C)	Melting temperature (°C)
PHB	15	175
PHBV	(−)1	136–162
LDPE	(−)81	105–110
PP	(−)7 to (−)35	160–168

a Abbreviations: PHB, polyhydroxybutyrate; PHBV, poly(3-hydroxybutyrate-*co*-3-hydroxyvalerate); LDPE, low-density poly(ethylene); PP, poly(propylene).

### 3.3 PHAs' mechanical properties

Based on the monomer units' composition, PHAs can show a wide range of mechanical properties from those of hard crystalline polyesters such as PHB to more elastic materials such as poly(3-hydroxyoctanoate) or PHO.^120,216^ Compared to PP, PHB exhibits similar tensile strength and modulus of elasticity, however, it has a significantly lower elongation at break (5–10%).^217,218^ This can be attributed to the cracks within the PHB spherulites that are created under conditions of non-externally applied stress.^175,176,219,220^ The addition of nucleating agents to the polymer melt during processing is one way to reduce PHB's brittleness.^179^ PHBV's mechanical properties depend on the molar ratio of HV.^122^ Generally, increasing HV's fraction lead to an increase in the toughness and flexibility of the copolymer, yet the tensile strength gradually decreases.^120^ Furthermore, when the HV composition is in the range of 30 to 60 mol%, it was observed that PHBV becomes very soft.^218^ A one-month analysis of PHBV showed that depending on the composition of the random copolymer, PHBV films can have a percentage elongation of more than 500%. Nonetheless, the PHBV films were found to exhibit this value for only a few days after the films were cast, after that it was observed that the copolymer can show brittleness with time.^217^ The embrittlement of both of PHB and PHBV polymers takes place during storage after initial crystallization from the melt. It is also believed that the reorganization of lamellar crystals produced during the initial crystallization process results from secondary crystallization. This leads to firmly restrict the amorphous polymer chains between the crystals.^175,221,222^ Polymer's stiffness is determined from the modulus of elasticity. The modulus of elasticity for PHAs ranges from the stiffer scl-PHA (3.5 × 10^3^ MPa) to the very ductile mcl-PHA (0.008 MPa).^172^ PHAs exhibit a wide range of elongation at break values (between 2% and 1000%).^192^ The tensile strength for PHAs usually ranges from 8.8 to 10^4^ MPa.^172^ The typical mechanical properties of PHB and PHBV along with other commercial plastics are shown in Table 20. Furthermore, Table 21 provides another overview of the mechanical and thermal properties of the synthetic plastics and PHA homo- and copolymers.

**Table 20:** Typical mechanical properties for PHAs along with other commercial polymers^120,122,222^

Polymer^a^	Tensile modulus (GPa)	Tensile strength (MPa)	Percentage elongation at break
PHB	1.7–3.5	40	3.0–6.0
PHBV	0.7–2.9	30–38	20
PLA	1.2–2.7	28–50	7.0–9.0
PCL	0.4	16.0	120–800
TPS	0.5–1.0^b^	2.6	47.0
PET	2.2	56.0	70–100
LDPE	0.2	10–15	300–500
PP	1.7	35–40	150
PS	1.6–3.1	12–50	3.0–4.0
PVC	0.3–2.4	10–60	12–32

a The values for mechanical properties will vary according to different factors such as, polymer crystallinity, molecular weight, orientation, as well as testing conditions.

b At low water content (5.0–7.0 wt%). Abbreviations: PHB, polyhydroxybutyrate; PHBV, poly(3-hydroxybutyrate-*co*-3-hydroxyvalerate); PLA, poly(lactic acid); PCL, poly(ε-caprolactone); TPS, thermoplastic starch; PET, poly(ethylene terephthalate); LDPE, low-density poly(ethylene); PP, poly(propylene); PS, poly(styrene); PVC, polyvinyl chloride.

**Table 21:** An overview of the mechanical and thermal properties of the synthetic plastics and PHA homo- and copolymers^222–224^^a^

Polymers	Mechanical properties		Thermal properties		
	Tensile strength (MPa)	Percentage elongation (%)	Crystallinity (%)	Melting temperature (°C)	Glass transition temperature (°C)
PHB	40	6	60	177	2
P4HB	104	1000	45	150	−51
PHBV	25	20	56	145	−7.25
PHBHHx	21	400	34	127	−1
P3HB-*co*-16 mol% 4-HB	26	444	NA	150	−7
P3HB-*co*-64 mol% 4-HB	17	591	15	50	−35
P3HB-*co*-78 mol% 4-HB	42	1120	17	49	−37
P3HB-*co*-82 mol% 4-HB	58	1320	18	52	−39
P3HB-*co*-90 mol% 4-HB	65	1080	28	50	−42
P3HB-*co*-3 mol% 3-HV	38	NA	NA	170	8
P3HB-*co*-9 mol% 3-HV	37	NA	NA	162	6
P3HB-*co*-14 mol% 3-HV	35	NA	NA	150	4
P3HB-*co*-20 mol% 3-HV	20	50	NA	145	−1
P3HB-*co*-25 mol% 3-HV	30	NA	NA	137	−6
P3HB-*co*-10 mol% 3HHx	21	NA	NA	127	−1
P3HB-*co*-15 mol% 3HHx	23	NA	NA	115	NA
P3HB-*co*-17 mol% 3HHx	20	NA	NA	120	NA
PP	38	400	50–70	176	−10
LDPE	10	620	35–55	130	−30
HDPE	19	576	NA	108–134	−110

a NA, not available. Abbreviations: PHB, polyhydroxybutyrate; P4HB, poly(4-hydroxybutyrate); PHBHHx, poly(3-hydroxybutyrate-*co*-3-hydroxyhexanoate); P3HB, poly(3-hydroxybutyrate); PP, poly(propylene); LDPE, low-density poly(ethylene); HDPE, high-density poly(ethylene).

### 3.4 PHAs' permeability & migration

The water vapor permeability for PHB and PHBV films have been reported to be similar to that of fossil-based thermoplastics such as polyvinyl chloride (PVC) or PET. This has attractive many food packaging industries to replace their packaging with PHB and PHBV films.^225–229^ In addition, PHAs have lower hydrophilicity than other biomaterials such as cellulose and starch and they are non-swelling.^225^ Since the degradation of PHAs polymers can be initiated by enzymatic or non-enzymatic hydrolysis, the solubility and diffusivity of water in PHAs become of great importance.^230–232^ Various studies in literature have reported water transport properties of PHB, PHBV films, as well as their blends with other biodegradable polymers under various conditions.^154,225–227,230,231,233–236^ Interestingly, it was found that the water sensitivity of other biopolymers can be reduced by the addition of PHAs. For instance, in one study, there was a drop of the water permeability of poly(vinyl alcohol) (PVA) films as a result of the incorporation of 10–50 wt% PHB.^236^ Furthermore, PHAs show low oxygen and CO_2_ permeability^193,194,237^ as well as they are found to exhibit good barrier properties against a number of organic solvents.^225,226,238^ The permeability of various organic liquids as well as vapors, water and CO_2_ through PHB films was investigated by Miguel *et al.*^225,239^ Results showed that the permeability was moderate to low for methanol, *n*-hexane, carbon tetrachloride and isopropyl ether while it was relatively high for moderately polar solvents such as chloroform, acetone and toluene. In another study, the water permeability of different PHA films was compared. Results suggested that water sorption and water vapor permeability in PHBV were virtually independent of the HV content in the range 0–24 wt%. This might be attributed to the similar crystallinity of the HB and HV segments.^227^ Other studies showed that PHBV demonstrates lower water permeability when compared to PHB and that the water vapor barrier increases with increasing HV content.^154,195^ This might be due to the slight decrease in crystallinity as the hydroxyvalerate content increases.^178^ In other studies, the water vapor permeability values for PHB were lower than PHBV.^238,240^ Moreover, many studies have discussed the role of crystallinity in determining PHAs' permeability properties. In one of these studies, the diffusion coefficient and equilibrium solubility of water molecules in PHB, polyglycolide (PGA), Skygreen® (styrene glycol, an aliphatic polyester of succinic acid/adipic acid-1,4-butanediol/ethylene glycol), PLLA, and PCL were explored. Results of the study suggested that the diffusion coefficients decreased in the order SG > PCL > PLLA > PHB > PGA. It is believed that this was partially attributed to variations in the crystallinity of these polymers.^230^ Poley *et al.* reported a CO_2_ diffusion coefficient value of 1 × 10^−9^ cm^2^ s^−1^ at 25 °C, which is slightly higher than what was reported earlier by Miguel *et al.* (4.4–4.7 × 10^−10^ cm^2^ s^−1^ at 30 °C). The oxygen diffusion coefficient was around 0.4 × 10^−9^ cm^2^ s^−1^ at 25 °C for PHB which slightly increased with increasing HV content (8–22 wt%). This increase was attributed to a reduction in crystallinity.^178,225^ The incorporation of inorganic laminar nanofillers such as clays can improve polymers' barrier properties. The addition of such nanofillers led to an increase in the tortuosity of the diffusion path.^240,241^ Studies have shown that a reduction in the oxygen permeability of PLA,^242–245^ PCL,^238,240^ PET,^246^ and PP^247,248^ was observed as a result of the addition of nano clays. Nonetheless, there have been a limited number of studies about the successful addition of nanofillers in enhancing the barrier properties of PHAs. In one of the studies, the thermal and barrier properties of organically modified kaolinite and OMMT in PHB-based nanocomposites prepared by melt blending with the addition of PCL as a plasticizer were compared. Results suggested an increase in gas, aroma and water vapor barrier performance for the nanocomposites. PHB- and PHB/PCL-based nanocomposites containing 4 wt% nano clay exhibited a reduction in oxygen permeability of a round 43% at 24 °C and 0% RH.^240^ In a similar study, the incorporation of 5 wt% OMMT resulted in around 20% and 27% reduction in oxygen permeability of PHB and PHBV films, respectively.^238^ Although a reduction in permeability is usually expected due to the formation of nanocomposite by layered clay silicates, the coexistence of phases with different permeabilities can lead to complex transport phenomena. On the one hand, an organophilic clay can increase superficial adsorption.^249–251^ A comparison of the permeability of PHB with PHBV and other polymers is presented in Table 22. Generally, PHB's and PHBV's barrier properties appear to be slightly better than those of PLA and potentially competitive with those of different synthetic plastics. Nonetheless, it is important to keep in mind that such data was collected using various measuring techniques and equipment. Moreover, there might be variations in terms of crystallinity and molecular weight of the tested polymers. Therefore, it is highly recommended to evaluate the barrier properties of individual PHAs for packaging of particular food types. When dealing with PHAs, migration becomes an important property. This is because the monomers or additives used during the fabrication of PHAs' based products may not be commonly used in conventional food contact materials. Therefore, there is always that concern that such materials can migrate into the packaged food. Bucci *et al.* investigated the total migration from PHB films into different food simulants, including distilled water, 3% acetic acid, 15% ethanol and *n*-heptane. The investigation was done for 10 days at 40 °C, with the exception of *n*-heptane where it was performed for 30 minutes at 20 °C. Results suggested that PHAs are considered safe for packaging of different food products. This conclusion was drawn as the total migration for all the simulants, was below the recommended limit of 8.0 mg dm^−2^ or 50 mg kg^−1^.^131^ One of the challenges with regards to the utilization of biodegradable polymers in the food packaging industry is the durability of the packaging with respect to the product shelf-life. Such a challenge makes the migration concern even more complex. During the storage of food products, it is crucial to avoid the environmental conditions that lead to packaging's degradation.^252,253^ Although neat PHB and PHBV are non-toxic, yet, there is a need for more information about the potential toxicity and migration behavior of degradation products produced during either processing or biodegradation.^179^ Furthermore, the potential migration of nanoparticles from PHA nanocomposite films into food products is another concern to be considered for future packaging applications. Such concerns are valid because nanoparticles are usually much more reactive than corresponding macroparticles. This can be attributed to the nanoparticles' large surface area which allows for a greater contact with cellular membranes. Moreover, it allows for a greater capacity for absorption and migration.^254^ In a study conducted by Šimon *et al.*, it was found that only very small particles that have a diameter of around 1 nm were able to migrate from the nanocomposites.^255^ The migration of specific minerals, namely iron, magnesium and silicon from biodegradable starch/nano clay nanocomposite films was studies by Avella *et al.* Results suggested that there was an insignificant trend in the levels of iron and magnesium in packaged vegetables. Nonetheless, a consistent increase in the amount of silicon was observed.^256^ In another study, the potential migration of Cloisite® 30B from PLA nanocomposite films was studied by Schmidt *et al.* Results suggested that there was no sign of any clay minerals migration, yet, migration of nanoparticles in the range of 50–800 nm were observed.^257^ Mauricio-Iglesias *et al.* suggested to monitor the specific migration properties of nanoparticles rather than the migration of their constituent elements.^258^ Overall, there should be a continuous need for risk evaluation due to any potential migration of nanoparticles from PHAs-based nanocomposite films or even degradation products from PHAs packaging materials.

**Table 22:** A comparison of the permeability of PHB with PHBV and other polymers^154,225,238,240,259^

Polymer^a^	Water vapor permeability^b^ (g mm m^−2^ day^−1^)	Oxygen permeability^c^ (ml mm m^−2^ day^−1^ atm^−1^)	Carbon dioxide permeability^c^ (ml mm m^−2^ day^−1^ atm^−1^)
PHB	1.0–5.0	2.0–10.0	3.0
PHBV	1.0–3.0	5.0–14.0	—
PLA	5.0–7.0	15.0–25.0	35–70
PCL	300	20–200	—
LDPE	0.5–2.0	50–200	800–1000
PET	0.5–2.0	1.0–5.0	15–20
PP	0.2–0.4	50–100	200–400
PS	1.0–4.0	100–150	250–500
PVC	1.0–2.0	2.0–8.0	10–15

a The values for permeability will vary according to different factors such as, polymer crystallinity, molecular weight, orientation, as well as testing conditions.

b At 23–38 °C, 50–90% relative humidity.

c At 23 °C, 0–50% relative humidity. Abbreviations: PHB, polyhydroxybutyrate; PHBV, poly(3-hydroxybutyrate-*co*-3-hydroxyvalerate); PLA, poly(lactic acid); PCL, poly(ε-caprolactone); LDPE, low-density poly(ethylene); PET, poly(ethylene terephthalate); PP, poly(propylene); PS, poly(styrene); PVC, polyvinyl chloride.

### 3.5 PHAs' degradation

There have been several studies about PHAs' biodegradation in the aerobic and anaerobic environments.^131,260,261^ PHAs are considered more readily biodegradable than PLA.^221,262^ PHAs' biodegradation includes biotic or abiotic hydrolysis followed by bio assimilation.^232^ The degradation process of intracellular PHAs takes place by the intracellular PHB depolymerase, which degrades PHAs into 3-HB.^263^ Different microorganisms can excrete extracellular PHA depolymerases that hydrolyze high molecular weight PHAs into water-soluble oligomers and monomers and consequently utilize these products as nutrients.^120^ Carbon dioxide and water are the resulted metabolic products under aerobic conditions,^231^ while methane can also be produced under anaerobic conditions.^264^ Therefore, throughout the degradation of PHAs, no harmful end products or intermediates are produced. In one study, the extracellular PHA depolymerase was subjected to purification from the different fungi and bacteria that are identified to degrade PHAs. Results suggested that *Penicillium*, *Cephalosporum*, *Paecilomyces* and *Trichoderma* were the dominant types of fungi while *Pseudomonas*, *Azotobacter*, *Bacillus* and *Streptomyces* were the dominant genera among bacteria.^265^ Different studies targeting the depolymerase mechanism of PHA have been reported in literature.^266–269^ According to Khanna and Srivastava, there are several factors that can affect PHAs' biodegradation rate. Such factors include, the crystallinity, molar mass, copolymer composition, chain mobility, hydrophilic/hydrophobic balance, and stereochemistry. Furthermore, environmental factors such as the temperature, pH, moisture, nutrient supply, and microbial population can also influence the biodegradation rate of PHAs.^120^ Different studies investigated the factors affecting PHAs' biodegradation rate in various mediums such as, sewage environments,^131,270^ marine environments,^271–273^ fresh water,^274,275^ compost media,^209,276^ and soil^232,265,273,277^ have been reported. Generally, lower degradation rate is associated with higher polymer crystallinity and melting point. Moreover, it was found that increasing the HV content in PHBV, can lead to a faster degradation.^231^ It was reported that PHBV degrades faster than PHB under aerobic conditions;^269,276–278^ yet, the opposite effect has been also reported.^260,261^ Several studies have investigated PHA-based nanocomposites' degradation rate. As a common observation, increasing the content of nanoparticles often leads to a lower PHB's or PHBV's biodegradation rate. Wang *et al.* have investigated the biodegradation rate of PHBV/OMMT in soil suspension. Results of the study showed that the biodegradation rate of the PHA-based nano composites decreases with increasing the content of OMMT. It is believed that such a reduction in the biodegradation rate is due to the formation of a tortuous path as a result of incorporating high aspect ratio nano clays into the PHA matrix. The tortuous path can restrict the penetration of microorganisms into the bulk of the material.^208,209^ Furthermore, the lower water permeability as well as the antimicrobial effect in some OMMTs may also lead to a decrease in the biodegradation rate.^195^ On the other hand, well-dispersed clay particles can lead to breakage of the polymer chains and hence an increase in the rate of degradation.^279^ In another study, an improvement in the rate of biodegradation of toughened PHB was achieved by incorporating titanate modified MMT. It is believed that the terminal hydroxylated edge groups of the silicate clay layers can absorb moisture from compost and act as initiation sites for polyester hydrolysis.^280^ Usually, any factor that increases PHAs' hydrolytic tendency will ultimately control its degradation.^251^ Maiti *et al.* reported a significant enhancement in the PHB's biodegradation rate due to the incorporation of 2 wt% organo-modified fluoromica. Results showed that in about seven weeks, an almost complete degradation was noticed. Furthermore, the study reported a significant drop in the biodegradation rate of neat PHB and PHB nanocomposites at higher temperatures. Such a decrease in the biodegradation rate might be attributed to the suppression of microorganisms at and above 60 °C. Another explanation of this drop might be associated to the increase in the crystallinity of these samples which is considered important as the amorphous regions are prone to hydrolysis followed by microorganism attack.^209^ With regards to food packaging, the performance of copolymer poly(HB-*co*-HV) Biopol®-coated paperboard trays overwrapped with a corn starch-based Mater-Bi® type ZF03U film was studied by Kantola and Helén. They have used organic tomato to be packed in the PHB-coated paperboard trays. Results suggested that the tomato stayed as fresh as those wrapped in perforated LDPE bags.^281^ Haugaard *et al.* investigated packaging of an orange juice simulant and a dressing in PHB cups. The study showed that the performance was as good as that of high-density polyethylene (HDPE) and superior when samples were stored under light.^282^ In another study, there was no substantial drop in the properties of PHB after subjecting it to the levels of gamma radiation required to sterilize food or packaging materials.^283^ By examining the mechanical, physical, dimensional as well as the sensorial analyses, PHB was concluded to be suitable for the storage of fat-rich products such as mayonnaise, margarine, and cream cheese.^135^ Furthermore, PHB was also found to be suitable for the packaging of sour cream.^284^ In addition, the impact of pasteurization on the packaging efficiency of PHB, PLA, PE, and PP films was examined by Levkane *et al.* The study claimed that sterilized PHB films could be efficiently used for the packaging of meat salad.^285^ A summary of some of PHAs' degradation studies in different environments along with their findings are shown in Table 23.^286^

**Table 23:** A summary of some of the PHAs' degradation studies^286^^a^

Polymer (co-monomer, %)	Medium/environment	Degradation method	Findings	References
PHA; in the form of bags	Compost	ASTM D5338	94% biodegradation after 180 days	287
PHBV (12% HV); in the form of films		ASTM D5338-15, mushroom compost	90% biodegradation after 200 days	288
PHB; in the form of 0.24 mm plate		ASTM D5338-98	99–100% mass loss after 112–140 days	289
PHB; in the form of 1.2 mm plate			98–100% mass loss after 84–112 days	
PHB; in the form of 5 mm plate			45% mass loss after 210 days	
PHB; in the form of 0.5 mm plate		ASTM D5929-96	100% mass loss after 182 days	
PHB; in the form of 1.2 mm plate				
PHB; in the form of 3.5 mm plate			94% mass loss after 350 days	
PHB; in the form of films		ISO 14855-1, compost factory organic waste	80% biodegradation after 110 days	290
PHBV (3% HV); in the form of films				
PHBV (20% HV); in the form of films			89% biodegradation after 110 days	
PHBV (40% HV); in the form of films			90% biodegradation after 110 days	
PH4B; in the form of films			90% biodegradation after 110 days	
PHB; in the form of films		ISO 14855-1, mature compost	80% biodegradation after 45 days	291
PHBV (3% HV); in the form of films			81% biodegradation after 45 days	
PHB; in the form of tensile samples		Non-ASTM, home composting	50% mass loss after 84 days	292
PHB; in the form of pellets		ISO 14855, mature organic municipal solid waste	92% mass loss after 78 days	293
PHB; in the form of 0.5 mm plate		ASTM D5929-96	100% mass loss after 182 days	289
PHB; in the form of 1.2 mm plate				
PHB; in the form of 3.5 mm plate			94% mass loss after 350 days	
PHBV (12% HV); in the form of films	Soil	ASTM D5988-12, agriculture field soil at 23–25 °C, 20% moisture	35% biodegradation after 200 days	288
PHB; in the form of nano-fiber films		Non-ASTM, fertile garden soil at 30 °C, 80% relative humidity, 10 cm depth	100% mass loss after 28 days	294
PHB; in the form of films		Non-ASTM, field soil at 21 °C and 28 °C, 50% moisture	After 35 days, 60% mass loss at 21 °C and 95% mass loss at 28 °C	295
PHBV (12% HV), in the form of films			After 35 days, 90% mass loss at 21 °C and 100% mass loss at 28 °C	
PHBHx (12% Hx); in the form of films			After 35 days, 92% mass loss at 21 °C and 100% mass loss at 28 °C	
PH4B (10% 4HB); in the form of films			After 35 days, 100% mass loss at 21 °C and 100% mass loss at 28 °C	
PHB; in the form of films		ASTM D5988, natural mature soil at 11–30 °C, 17–23% moisture	60% mass loss after 112 days	296
PHBV (8% HV); in the form of films				
PHB; in the form of films		ASTM D5988-03, commercial soil at 23 °C, 33% moisture	82% mass loss after 80 days	297
PHA; in the form of films			76% mass loss after 80 days	
PHA; in the form of films		ASTM D5988-03, a mixture of topsoil, farm soil and sand at 20 °C, 60% of water holding capacity	70% biodegradation after 660 days	298
PHA; in the form of films		Non-ASTM, farmland topsoil at 35% moisture	32% mass loss after 140 days	299
PHA; in the form of films			33% mass loss after 60 days	300
PHA; in the form of films		Non-ASTM, farmland topsoil at 25 °C, 35% moisture	35% mass loss after 120 days	301
PHA; in the form of films		Non-ASTM, farmland topsoil at 30–40% moisture	22% mass loss after 60 days	302
PHB; in the form of films	Marine	Non-ASTM, eutrophic reservoir at 18–25 °C	43.5% mass loss after 42 days	303
PHB; in the form of solid		Non-ASTM, South China sea at 27–30 °C	62% mass loss after 160 days	304
PHB; in the form of films			58% mass loss after 160 days	
PHBV (11% HV); in the form of solid			87% mass loss after 160 days	
PHBV (11% HV); in the form of films			54% mass loss after 160 days	
PHB; in the form of films		ASTM D6691, woods hole harbor water at 30 °C	90% mass loss after 100 days	154
PH4B (44% 4HB); in the form of films			80% mineralization after 100 days	
PH4B (47% 4HB); in the form of films			82% mineralization after 100 days	
PHBV (8% HV); in the form of films			85% mineralization after 100 days	
PHBV (12% HV); in the form of films			100% mineralization after 100 days	
PHBV (8% HV); in the form of films		Non-ASTM, Lorient harbour at 25 °C	36% mass loss after 180 days	305
PHBV (8% HV); in the form of powder		Non-ASTM, Lorient harbour water + foreshore sand at 25 °C	90% biodegradation after 210 days	
PHA 2200; in the form of films		ASTM D6691-09 at 30 °C	52% biodegradation after 365 days	306
PHA 4100; in the form of films			82% biodegradation after 365 days	
PHBV (12% HV); in the form of films		Non-ASTM, Baltic sea water at 17–20 °C	60% mass loss after 42 days	307
PHBHx (6.5% HV); in the form of films		Non-ASTM, coastal sea water at 23 °C	89% biodegradation after 148 days	308
PHBHx (7.1% HV); in the form of films			55% biodegradation and 77% biodegradation after removal of the outlier in the data for the sample after 195 days	
PHA; in the form of films		Non-ASTM, tropical river water at 28 °C	71% mass loss after 86 days	309
PHBHx (11% HV); in the form of films		Non-ASTM, sea water at 27 °C	35% biodegradation after 28 days	310

a Abbreviations: PHA, polyhydroxyalcanoates; PHBV, poly(3-hydroxybutyrate-*co*-3-hydroxyvalerate); PHB, polyhydroxybutyrate; PH4B, poly(3-hydroxybutyrate-*co*-4-hydroxybutyrate); PHBHx, poly(3-hydroxybutyrate-*co*-3-hydroxyhexanoate); PHA 2200; Mirel™ PHA plastic film; PHA 4100; Mirel™ PHA plastic film.

## 4. Applications

**Fig. 40:**
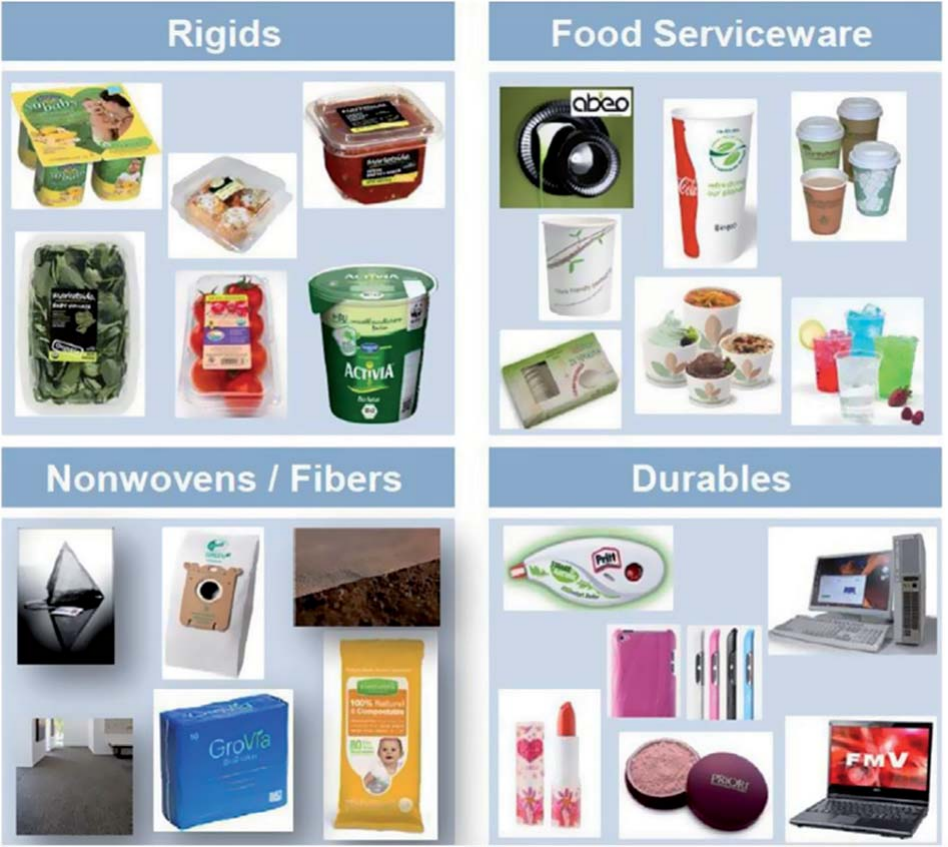
Examples of PLA's applications.^24^

**Fig. 41:**
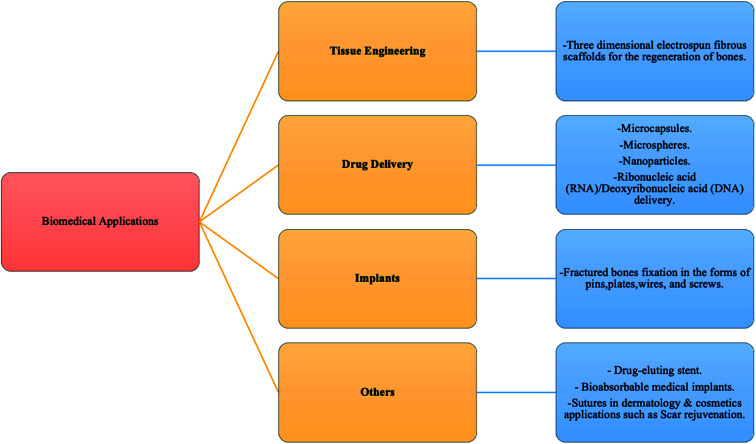
Some of PLA's biomedical applications.^62^

PLA has found acceptance from consumers due to its availability and flexibility. Moreover, different polymer products can be fabricated using PLA which makes it a tempted substitute for petroleum-based materials such as PE, PS, or PP. Behaving like PET and performing like PP, PLA is considered very versatile. PLA can be used in various applications due to its ability to be thermally and stressed crystalized, copolymerized, as well as impact modified. Some of the applications where PLA has been used recently are shown in Fig. 40. Due to its outstanding organoleptic characteristics, PLA is considered as an attractive alternative for food packaging and food contact applications. PLA can be injection blow molded preforms for bottles use, and at the same time can be formed into transparent films, and fibers.^24^ Comparing it with petroleum based polymers, PLA offers reasonably good physical, mechanical, optical, and barrier properties.^311^ Comparable to polystyrene, PLA offers a medium water and oxygen permeability level.^46,81,312^ The coefficients of permeability of oxygen, nitrogen, water, and carbon dioxide for PLA are higher than PET but lower than PS.^311,313,314^ Randomly oriented PLA has good stiffness and strength; however, it tends to be brittle. Oriented PLA's performance surpasses the oriented PS's performance but it is comparable to the performance of oriented PET.^315^ The elongation at break as well as the Izod impact strength of PLA is lower than those of PP, PS, and HDPE, nonetheless, its flexural and tensile properties are higher.^316^ Mechanical, thermal, and barrier properties depend on the optical purity of PLA.^39,75,317–321^ With a reasonable price, PLA is the largest produced biodegradable polymer in the world. Therefore, several food industries, especially those involving single-use applications like food packaging, are starting to utilize PLA as a food and drinks packaging material. PLA is also used for hygienic products for nonwoven materials. PLA is sealable and printable. Due to its bio resorption and biocompatibility characteristics, PLA is considered as a good alternative to be used for orthopedic fixations (screws, pins), suture threads and clips, and resorbable implants. Fig. 41 summarizes some of the biomedical applications of PLA.^62^

**Fig. 42:**
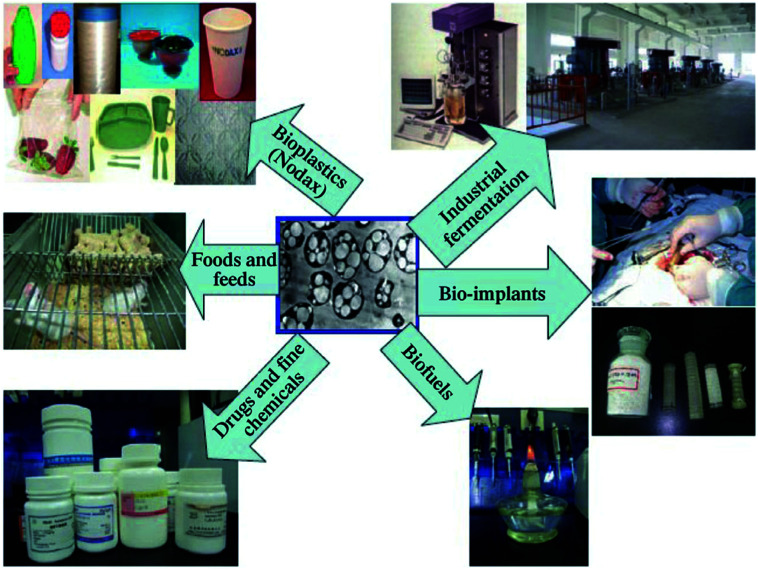
Some of PHAs' applications.^24^

PHAs demonstrate good barrier properties against oxygen and a slightly higher barrier effect against water vapor compared to other types of biopolymers. PHAs can be used in different applications ranging from commercial to medical.^322^ As illustrated in Fig. 42, PHAs are used in food, biofuels, drugs, fine chemicals, bioplastics, industrial fermentation, and bio-implants. They can be also used in disposable items such as diapers, cosmetic containers, razors, cups, feminine hygiene products, and utensils. They can also be utilized as packaging films in containers, paper coatings, and shopping bags. Furthermore, they have been used efficiently as medical surgical garments, compostable bags, lids, upholstery, carpet and thermoforming tubs.^24^ PHB has been also reportedly used in food and medicine packaging and agriculture. Both: the biodegradability and the biocompatibility characteristics made PHBV an outstanding material with a wide range of applications in various sectors. PHBV's excellent properties such as its low cytotoxicity, biological origin, absorption capacity, piezoelectricity, thermo-plasticity, high degree of crystallinity, and its resistance to ultraviolet radiation and acceptable amounts of alcohols, fats, and oils have made this copolymer to become very promising for biomedical applications.^133,291,323–326^ Moreover, PHBV exhibits chemical inactivity, excellent oxygen barrier properties, high viscosity in a liquid state-an aspect that is favorable in extrusion processes, and better mechanical properties, such as an increase in surface tension and greater flexibility compared to PHB.^327^ Due to its biotechnological potential, and its applicability in the medical, agricultural, and packaging fields, PHBV has recently attracted the attention of both industry and researchers as a promising material.^328,329^ The challenges and difficulties that patients of advanced age experience are a key factor that determine the raising demand for bio-degradable polymers. Today, there are various medical treatments that are dependent upon PHAs.^330,331^ For example, PHAs are used today in drug release and transport systems,^324,325^ absorbable surgical sutures, medical packaging,^326^ and in the fabrication of cardiovascular stents.^323^ They are also used in the field of tissue engineering in applications that include biosensors, the elaboration of tissue patches, and biodegradable implants. Furthermore, PHBV is used in the fabrication of porous scaffolds that allow the treatment of bone defects caused by diseases or injuries where conventional treatments are unsuccessful.^332–335^ Outside the biomedical field, PHBV has been used in daily disposable objects such as cosmetics, packaging, containers, bags, as well as hygiene products (towels, diapers, and handkerchiefs). PHBV has been successfully used in products requiring high mechanical resistance, such as, printed wiring boards (for electronics), different car panels, and helmets for cyclists.^317,336–338^

## 5. Current challenges

**Fig. 43:**
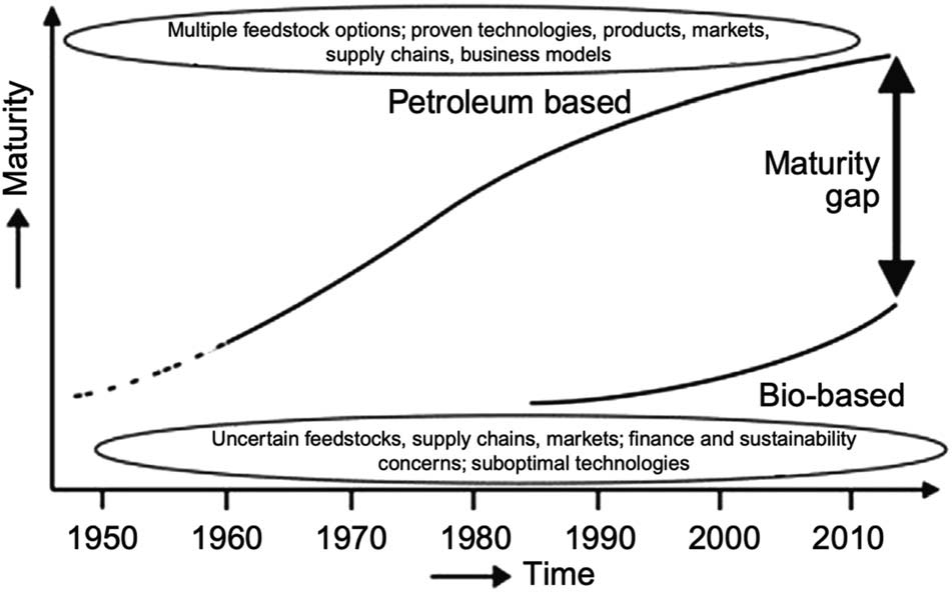
The gap between conventional and bioplastics.^24,342^

Bioplastics today are facing many challenges in order to become commercially viable. Some of these challenges are cost feasibility, sustainably grown biomass, lack of adequate labeling, composting programs and infrastructure, as well as concerns over contamination of recycling systems. Furthermore, the widespread use of biopolymers is hindered due to high costs including high investment processing costs, poor performance characteristics. Moreover, the pressure that some bioplastics such as PLA add on agricultural crops to continuously satisfy the requirements of the ever-growing population is another reason that limits the widespread use of bioplastics. In addition, the use of PHAs in various applications today remains also restricted despite its excellent properties and characteristics. This is mainly attributed to their high production cost. Therefore, the utilization of PHAs is only economically feasible for specific applications.^119,173^ Thus, the focus of the research today is on developing much more efficient fermentative routes with renewable sources as a substrate,^339^ finding new microbial strains that are able to accumulate higher levels of PHAs,^340^ and reducing the costs associated with the polymer extraction process.^341^ A comparison between the status of petroleum-conventional based polymers and bio-based polymers is presented in Fig. 43. As seen in the figure, there is still a considerable gap for a real market realization of biopolymers. Biopolymers are yet to establish proven technology to drive growth through innovation and build reliable supply and customer chains.^24,342^

## 6. Conclusions, future research & outlook

The main purpose for this work is to provide an overview on state of the art of the research activities on PLA's and PHAs' physical, thermal, rheological, and mechanical properties. Moreover, permeability as well as migration properties for both bio-based plastics have been discussed. In addition, PLA's recyclability, sustainability, and environmental assessment have been also reviewed. Different applications that both biopolymers can replace petroleum-based plastics in have been highlighted. In order for PLA and PHAs to replace petroleum-based plastics, they still need to go through many developments, innovation, and research in many ways. One way that is still under debate is the environmental friendliness of PLA products. This is because the agricultural activities accompanied with the production of PLA can lead to the emissions of carbon as well as other sources of pollution such as water source pollution and the consumption of fossil fuel for electricity. Therefore, one way to look for in the future is the reduction of the emissions of carbon during the production of PLA. A successful reduction of such emissions and pollutions that are typically accompanied by the production of PLA will increase its feasibility and give it the lead in the race against petroleum-based polymers. Before expanding PHAs' production, there is still a need to study their migration properties as well as the migration behavior of the nanofiller, nanoparticles, and nano clays incorporated with PHAs. This is of great importance and shall be demonstrated before approving to expand the production of PHA-based nanocomposites on a commercial scale in food packaging. Moreover, more studies about PHA-based multilayer films and active packaging are required. New production techniques are required to produce PHAs in a less expensive cost, especially now with the increasing price of oil. Therefore, optimum cost production methods can aid in narrowing the price gap between the petroleum-based plastics and biodegradable PHAs. Furthermore, new processing techniques are required so as to increase PHAs' thermal stability and toughness. Finally, PHAs can be a strong candidate for food packaging applications if more efforts are put together towards further analysis and investigations. Work on the review of the effect of different plasticizers, nucleating agents, as well as blends with other biodegradable polymers on the properties of both polymers is in progress.

## Conflicts of interest

Each of the authors confirms that this manuscript has not been previously published and is not currently under consideration by any other journal. To the best of our knowledge, none of the authors have any conflict of interest, financial or otherwise.

## Supplementary Material
